# Modeling Heterogeneity in the Long-Term Trajectories of Individuals’ Well-Being

**DOI:** 10.1177/01461672251331654

**Published:** 2025-04-29

**Authors:** Robin Wollast, Joseph B. Phillips, Chloe Bracegirdle, Olivia Spiegler, Chris G. Sibley, Éric Lacourse, Nikhil K. Sengupta

**Affiliations:** 1 University of Kent, Canterbury, UK; 2 Cardiff University, Wales, UK; 3 University of Oxford, UK; 4 University of Applied Sciences, Göttingen, Germany; 5 The University of Auckland, New Zealand; 6 Université de Montréal, QC, Canada

**Keywords:** well-being, belongingness, social support, self-esteem, life satisfaction

## Abstract

Very little is known about how long-term well-being trajectories vary across populations. Using data from 45,160 adults in New Zealand (62% women, *M*_age_ = 41 years) surveyed annually over 13 years, we identified latent trajectories for belongingness, social support, self-esteem, and life satisfaction. Through a group-based trajectory modeling approach, we found five trajectory groups: low (3%–5%), moderate (11%–17%), moderate-high (29%–32%), high (35%–45%), and very high (11%–20%) well-being. While most individuals showed minimal changes, those with initially low well-being experienced the greatest change, in the direction of decreasing well-being over time. Individuals with higher education were more likely to follow higher well-being trajectories. Similarly, women were more likely to follow higher well-being trajectories, except for self-esteem, where men tended to score higher over time. Lastly, age and ethnicity demonstrated more complex patterns. These findings highlight the importance of acknowledging long-term heterogeneity in well-being trajectories and emphasize the need for targeted preventive mental health interventions, particularly for individuals who begin with lower well-being levels.

Well-being is a multifaceted construct encompassing people’s appraisals of themselves (Paradise & Kernis, 2002), their lives (Diener et al., 2002), and their social connectedness (Verhagen et al., 2018). The vast empirical literature on well-being in psychology has demonstrated the heterogeneity of the construct by revealing its different facets, how they differ between people, and what predicts those between-person differences (Quevedo & Abella, 2011; Trudel-Fitzgerald et al., 2021). It provides relatively less information on temporal heterogeneity—how well-being differs *within* people over time. The longitudinal studies that do exist can only characterize the overall trajectory of a given well-being indicator across a given population, showing evidence of both stability and change (Wunder et al., 2013). This does not tell us whether the trajectories themselves are heterogenous, with different groups in the population experiencing different rates of change over time (see Hsu, 2012 for a notable exception). For example, depending on people’s prior levels of well-being, they may experience more or less stable levels in the future, and any instability may be in the positive or negative direction (or indeed, both, at different phases of their lives). Person-centered, group-based trajectory modeling can reveal these complex temporal dynamics by identifying latent clusters in the population that follow differently shaped trajectories. This method can also tell us whether members of particular social categories, especially those most vulnerable, such as people with lower levels of education, are more likely to follow one trajectory versus another. However, the large-scale and long-term longitudinal data required for this approach are relatively rare. Here, we address this gap by leveraging a 13-wave longitudinal probability sample of New Zealand adults (*N* = 45,160), surveyed annually over 13 years, to model the potentially heterogeneous long-term trajectories of well-being, using four different indicators: felt belongingness, social support, self-esteem, and life satisfaction.

Felt belongingness encompasses the feeling of acceptance, inclusion, and value by others, providing individuals with a sense of being an integral part of their social environment (Allen et al., 2021). Social support captures the perception and availability of resources, assistance, and emotional comfort provided by social networks, including family, friends, and the community (Taylor, 2011). Self-esteem reflects individuals’ overall evaluation and perception of their own worth, value, and competence. It involves beliefs and feelings about one’s abilities, achievements, and self-worth (Pyszczynski et al., 2004). Life satisfaction is a global assessment of individuals’ overall evaluation of their life, including cognitive judgments and affective experiences of happiness and fulfillment. It reflects individuals’ subjective appraisal of the extent to which their aspirations, goals, and desires have been achieved or met (Pavot & Dierner, 2008). Each of these variables is considered a key indicator of overall mental health (e.g., Buecker et al., 2023; Paradise and Kernis, 2002). Understanding how different subgroups in the population differ in their trajectories of these four well-being facets will provide important information about who might be most at risk of experiencing worsening mental health outcomes over time.

## Focusing on Changes (In Addition to Differences) in Well-Being over Time

Examining well-being with cross-sectional methods capture valuable between-person differences at a specific point in time (e.g., Bleidorn et al., 2016; Joshanloo, 2018; Painter, 2013; Stone et al., 2010) but fails to pick up a number of key sources of temporal heterogeneity. The first is the general direction of well-being over time (e.g., stability, improvements, declines). Cross-sectional comparisons between those with low and high well-being may be comparing individuals low but improving in their well-being with individuals high but declining in their well-being. The second source of temporal heterogeneity is different levels of stability and volatility (e.g., sharp or gradual declines). This could reflect individual circumstances such as employment (Howard et al., 2022), as well as different levels of responsiveness to changes in life circumstances (Tiet et al., 1998).

Longitudinal studies have investigated the temporal dynamics of well-being. Descriptive and inferential analyses, in addition to meta-analyses, have found that life satisfaction and positive and negative affect tend to fluctuate over time (Buecker et al., 2023; FitzRoy & Nolan, 2020; Gadermann & Zumbo, 2007), while self-esteem may increase in adolescence and young adulthood but decline in old age (Erol & Orth, 2011; Orth et al., 2015, 2018). Inferential analyses also find that social support tends to lead to downstream increases in a variety of other well-being indicators over extended periods of time (Burton-Jeangros & Zimmermann-Sloutskis, 2016; Galambos et al., 2006; Hsu, 2012; Marshall et al., 2014). However, with few exceptions (e.g., Hsu, 2012), these studies characterize overall well-being trajectories, including individual-level variables that affect the level of well-being and deviations from the overall trajectory. This may hide substantial heterogeneity across individuals caused by unobserved individual-level variables. Indeed, a recent review of the literature on subjective well-being called for more studies utilizing longitudinal designs and analysis methods that can identify the heterogeneity underlying average trends (Diener et al., 2018). Such an approach can reveal important information, for example, Mancini et al. (2011) demonstrated meaningful heterogeneity in well-being trajectories in response to major life events such as bereavement, divorce, and marriage. Past studies also tend to use one indicator of well-being or another, which opens up the possibility that the results only pertain to one specific component of well-being.

To move beyond the estimation of a single trajectory, one can employ a person-oriented group-based trajectory modeling approach (Jones & Nagin, 2007; van der Nest et al., 2020). This modeling technique effectively captures the complexity of longitudinal patterns by identifying clusters of individuals with similar trajectories over time (Nagin, 2010). This flexible approach has the advantage of mapping not only average trends but also different trajectories within a population. This enables the exploration of hidden heterogeneity, which can help target tailored support for vulnerable groups and provide a better understanding of individuals’ developmental changes over time.

## Different Forms of Heterogeneity

**Figure 1.**
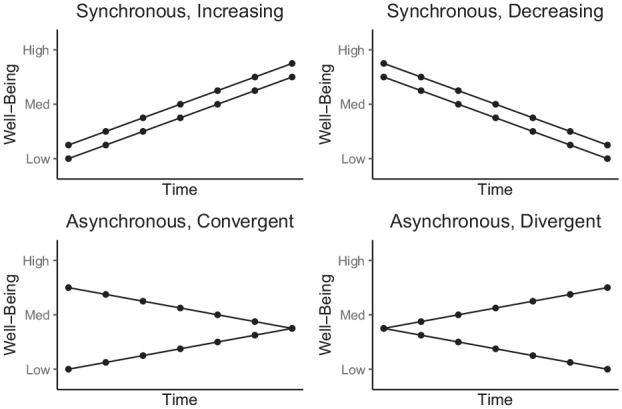
Hypothetical trajectory types.

If there is hidden heterogeneity in well-being trajectories over time, there are different forms that it might take, each with different theoretical and practical implications (see Figure 1). For example, people with different levels of well-being may increase or decrease about the same amount over time, but with each following their own unique trajectory based on their initial level of well-being. This pattern is referred to as *synchronous trajectories* (Le Blanc & Kaspy, 1998). Synchronous trajectories provide limited incremental information to guide theory or practice, as they suggest that people’s future level of well-being tracks with their prior level of well-being. Nonetheless, the trajectories themselves are still able to reveal the degree of heterogeneity in the population (about which we currently know very little) by telling us what proportions of people fall into higher or lower trajectories as well as the predictors of trajectory membership (see sections below).

There are also several different ways in which trajectories may be asynchronous or nonsynchronous (Le Blanc & Kaspy, 1998). First, people who start off high may report progressively lower well-being over time, whereas people who start off low may report progressively higher well-being over time. We call this pattern *Convergent Trajectories*. Convergent trajectories would imply that different subgroups in the population become more similar in their well-being over time, regardless of where they start off. This could indicate regression to a population-level mean, and once again, would have limited theoretical or practical applicability.

However, it would tell us more than the synchronous trajectories would. For example, it would imply that the logic of the “hedonic treadmill” theory of well-being (Diener et al., 2006), in which each person has a set point of well-being that they return to over time, can be applied to the population level. That is, each population has a set point of well-being, and all clusters within a population tend toward that level over time. Practically, this would suggest it may not be particularly useful to identify subpopulations who may be more or less vulnerable. Instead, in this case, policymakers would be advised to prioritize increasing the set point of well-being across their target population as a whole, which would ultimately benefit everyone regardless of their current well-being level.

A second type of asynchronous trajectory would be one in which people who start off at a similar level of well-being may follow increasingly different trajectories over time, with some individuals experiencing rising well-being while others experience declining well-being. We call this pattern *Divergent Trajectories*. This pattern would have the most immediate theoretical and practical relevance. Theoretically, it would imply that the hedonic treadmill view of well-being (Diener et al., 2006) is not entirely accurate. Practically, it would imply that interventions to target well-being should be especially sensitive to the risk of people with the lowest levels of well-being experiencing increasingly worse outcomes over time.

We also sought to uncover whether the trajectories of our selected well-being indicators (i.e., felt belongingness, social support, self-esteem, and life satisfaction) followed similar or different patterns over time. Previous longitudinal research shows that these well-being indicators are positively correlated (e.g., Galambos et al., 2006; Howard et al., 2022), suggesting they will follow similar trajectories over time. Other studies indicate that the correlations between different indicators may differ across populations. For example, Diener and Diener (1995) found that self-esteem and life satisfaction were consistently positively correlated, but the association was stronger in individualistic than collectivist societies. Overall, we expect our four well-being indicators to follow similar trajectories in the New Zealand context.

## Demographic Differences in Well-Being

Differences in levels of education significantly shape individuals’ experiences of felt belongingness, social support, self-esteem, and life satisfaction. People with high levels of education benefit from access to diverse social networks and intellectual engagement opportunities facilitated by their educational attainment (Frei & Axhausen, 2007). They also enjoy a stronger support system, comprising peers and mentors, contributing to higher levels of social support (Frei & Axhausen, 2007). Moreover, individuals with higher education often demonstrate higher levels of self-esteem, as their educational achievements boost their confidence and provide a sense of accomplishment (De Araujo & Lagos, 2013). Regarding life satisfaction, higher-educated individuals generally report greater levels, which can be attributed to increased economic opportunities, better job prospects, and an overall better quality of life (Joshanloo, 2018).

Age differences also exert a significant influence on well-being. Adolescents typically report lower levels of felt belongingness than adults (Painter, 2013). There is a general dearth of research regarding how social support varies with age. However, adolescence and early adulthood involve navigating identity formation and establishing social connections. This can entail some level of instability in individuals’ sense of self and level of social connection (Kroger, 2006; Wrzus et al., 2013), reducing felt belongingness in a given setting. Older adults tend to show higher levels of felt belongingness, benefiting from the time invested in cultivating fewer, but deeper and stable social relationships and establishing a sense of community (Wrzus et al., 2013). Furthermore, self-esteem tends to increase with age, as older individuals often develop a stronger sense of self-acceptance and experience fewer concerns about social comparison (Bleidorn et al., 2016; Kroger, 2006). Finally, numerous studies have consistently shown that life satisfaction demonstrates a U-shaped pattern, with higher levels reported among both younger and older adults compared to individuals in middle-age (Blanchflower & Oswald, 2008; Stone et al., 2010; see also Buecker et al., 2023).

Gender differences also play an essential role in influencing individuals’ well-being (Wollast et al., 2025). Research suggests that women often express a greater sense of belongingness, indicating a stronger feeling of connection and inclusion within social groups (Hagerty et al., 1992; McLaren et al., 2001, but see also Rainey et al., 2018). Similarly, women provide more social support to others, draw on socially supportive networks more consistently, and may benefit more from social support (Taylor et al., 2000; but see also Wollast et al., 2024). However, women may face unique challenges and societal expectations that can negatively impact their self-esteem, often experiencing higher levels of self-criticism and lower self-worth compared to men (Bleidorn et al., 2016; Wollast et al., 2020). Despite these challenges, women tend to report similar or slightly higher levels of life satisfaction (Batz-Barbarich et al., 2018), potentially due to their ability to maintain and foster strong social connections.

Similarly, ethnicity has been found to be associated with well-being. Indeed, ethnic minorities often encounter prejudice and discrimination, resulting in diminished well-being (Benner et al., 2022; Vines et al., 2017). Several longitudinal studies of self-esteem in U.S. samples (Erol & Orth, 2011; Orth et al., 2010; Shaw et al., 2010) have suggested that minoritized ethnic groups experience a stronger self-esteem increase in youth but a stronger decrease in old age, compared to the ethnic majority. However, these ethnic differences were not observed in a recent meta-analysis (Orth et al., 2018). Our exploration of a diverse sample in New Zealand, including Europeans, Māori, Asian, and Pacific people, aims to understand the temporal dynamics of well-being trajectories among these groups. This approach will help identify potential disparities and contribute to the development of targeted interventions promoting equitable outcomes across the spectrum of ethnic backgrounds. Furthermore, most prior studies are cross-sectional and can therefore only identify whether there are differences in well-being at a specific point in time depending on ethnicity, age, gender, and education. By assessing well-being trajectories over 13 years, we can additionally identify whether demographic differences are associated with greater improvements, declines, or stability in well-being over time. For example, perhaps the ongoing prejudice and discrimination often experienced by ethnic minorities leads not only to lower levels of well-being, but also to persistent decreases in well-being over time.

## The Current Investigation

This study analyzes the heterogeneity in well-being over a period of 13 years in a New Zealand sample using 13 waves of data collection from 2009 to 2021. We aim to identify clusters of well-being trajectories that differ in terms of level (i.e., low, moderate, high), direction (increasing, stable, decreasing), and shape (i.e., constant, linear, quadratic, or cubic). To achieve this, we conduct a person-oriented group-based trajectory modeling from a latent class growth analysis (Nagin, 2010; van der Nest et al., 2020) to identify homogeneous subgroups of well-being trajectories within the larger heterogeneous population. To test whether different well-being indicators follow similar or different patterns, we employ a variety of indicators of well-being, including felt belongingness, social support, self-esteem, and life satisfaction. Our goal in this research was to address three research questions:

(1) Are there subgroups within the New Zealand population that show distinct trajectories of well-being over time? We expect that trajectories will differ based on initial levels of felt belongingness, social support, self-esteem, and life satisfaction. This expectation is partly based on strong between-person variation in cross-sectional studies. We additionally base this expectation on past theorizing that expects variables like self-esteem and life satisfaction to be mostly stable over time, save for drastic changes in life circumstances. Owing to the scarcity of prior research, we lack specific expectations regarding the shape of different trajectories. However, we will investigate whether their overall pattern follows a synchronous (increasing vs. decreasing) or asynchronous (convergent vs. divergent) pattern.
(2) Are trajectories of felt belongingness, social support, self-esteem, and life satisfaction interconnected? On the one hand, in the studies where these indicators are examined in tandem, they tend to be highly correlated with one another (e.g., Howard et al., 2022). Hence, we expect trajectories in one variable to look similar to other variables. In other words, someone who experiences a trajectory of self-esteem that begins at a low level and linearly decreases will also demonstrate life satisfaction that begins low and linearly decreases. However, through testing multiple indicators, we leave open the possibility that different indicators of well-being show varying starting values and degrees of stability and linearity.
(3) How predictive are demographic characteristics of well-being trajectory membership? We expect that people with lower levels of education would be more likely to follow trajectories characterized by low levels on the four well-being indicators, over time. Additionally, we expected that younger people would be more likely to follow trajectories characterized by low levels of felt belongingness and self-esteem, along with more volatile patterns of life satisfaction. We lack specific expectations regarding the changes in social support with age. However, we might imagine that social support may decline among older adults, who are retired and gradually withdrawing from social roles (Kroger, 2006; Wrzus et al., 2013). In addition, we expect that women would be more likely to follow trajectories characterized by high levels of felt belongingness, social support, and life satisfaction but low levels of self-esteem over time, as compared to men. Finally, we hypothesize that members of ethnic minority groups will be overrepresented in trajectories of low well-being.

## Methods

The New Zealand Attitudes and Values Study was approved by the University of Auckland Human Participants Ethics Committee on May 26, 2021 until May 26, 2024, and renewed on May 02, 2023 until May 26, 2027. Reference Number: UAHPEC22576. All participants gave informed consent. The data described in the paper are part of New Zealand Attitudes and Values Survey (NZAVS). Full copies of the NZAVS data files are held by all members of the NZAVS management team and advisory board. A de-identified dataset containing the variables analyzed in this manuscript is available upon request from the corresponding author, or any member of the NZAVS advisory board for the purposes of replication or checking of any published study using NZAVS data. The SAS syntax and related materials used to test all models reported in this manuscript are available on the NZAVS OSF: https://files.osf.io/v1/resources/75snb/providers/osfstorage/6656259265e1de458e893ca7/?zip=

### Participants

The final longitudinal sample comprised 45,160 participants (out of a total of 69,207) who completed at least 3 waves of the NZAVS between 2009 and 2021 (62% women, *M*_age_ = 40.66, *SD*_age_ = 14.41, at Wave 1). The participants’ ethnic distribution reveals a predominant European representation at 77.0%, followed by Māori at 15.5%, Asian at 4.0%, and Pacific at 3.5%.

The NZAVS is an ongoing study that has been conducting an annual longitudinal panel survey of adult New Zealanders since 2009. Data collection occurs year-round, but respondents fill out surveys once every 12 months. The data included here were collected starting in October 2009 (Wave 1) until September 2021 (end of Wave 13). See Sibley (2021) for full details regarding NZAVS sampling and methodology.

### Missing Values and Dropout Rates

In line with the primary method of handling missing data with group-based trajectory modeling (Jones et al., 2001; Nagin, 1999), we use Full Information Maximum Likelihood method. Figure S1 reports the total number of waves of data collection completed per participant and Figure S2 reports indicators associated with the attrition rate (available in the online Supplemental Material).

### Measures

Items were embedded within the larger NZAVS questionnaire battery, and as such were based on short-form scales due to space constraints. Participants provided sociodemographic information including education, age, gender, and ethnicity as well as psychological variables. Table S1 reports the McDonald Omega coefficient or inter-items correlations for all variables for each wave of data collection. Importantly, the majority of short-form scales employed by the NZAVS demonstrate adequate composite reliability compared to their original full-form versions (Sibley et al., 2024).

#### Felt Belongingness

Felt belongingness was measured using three items adapted from the Sense of Belonging Instrument developed by Hagerty and Patusky (1995). These items were: “I know that people in my life accept and value me,” “I feel like an outsider” (reverse-coded), and “I know that people around me share my attitudes and beliefs.” Items were rated on a scale ranging from 1 (*Strongly disagree*) to 7 (*Strongly agree*). Composite reliability was low (ω_range_ = .56 − .64, see Table S1).

#### Social Support

Social support was measured using three items adapted from Cutrona and Russell’s (1987) Social Provisions Scale. These items were: “There are people I can depend on to help me if I really need it,” “There is no one I can turn to for guidance in times of stress” (reverse-coded), and “I know there are people I can turn to when I need help.” Items were rated on a scale ranging from 1 (*Very inaccurate*) to 7 (*Very accurate*). Composite reliability ranged from acceptable to good (ω_range_ = .75 − .84, see Table S1).

#### Self-Esteem

Self-esteem was measured using three items adapted from Rosenberg’s (1965) Self-Esteem Scale. These items were: “On the whole I am satisfied with myself,” “I take a positive attitude toward myself,” and “I am inclined to feel that I am a failure” (reverse-coded). Items were rated on a scale ranging from 1 (*Very inaccurate*) to 7 (*Very accurate*). Composite reliability ranged from acceptable to good (ω_range_ = .76 − .83, see Table S1).

#### Life Satisfaction

Life satisfaction was measured using two items adapted from the Satisfaction with Life Scale (Diener et al., 1985). These items were: “I am satisfied with my life” and “In most ways my life is close to ideal.” Items were rated on a scale ranging from 1 (*Strongly disagree*) to 7 (*Strongly agree*). The correlations between items were high (*r*_range_ = .64 − .70, see Table S1).

## Statistical Modeling

**Figure 2.**
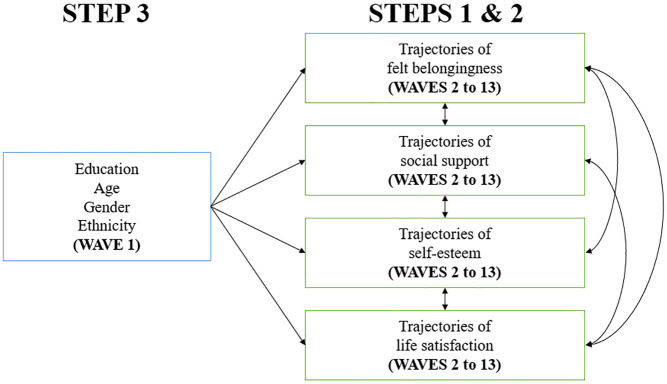
Data analysis plan.

The statistical analyses were divided into three steps: (1) identifying distinct trajectories within the sample of felt belongingness, social support, self-esteem, and life satisfaction, (2) investigating the co-occurrence (i.e., associations) of these trajectories over time, and (3) identifying the demographic variables (i.e., education, age, gender, ethnicity) distinguishing these trajectories. For this study, education was collected at Wave 1 (2009), gender, ethnicity, and age were collected throughout the study, but age was adjusted to Wave 1. Trajectories of well-being were modeled from Wave 2 (2010) to Wave 13 (2021). All statistical analyses were performed using RStudio (RStudio Team, 2023), SAS 9.4 (SAS Institute, Inc., Cary, NC, USA, 2016), and SPSS Statistics 29 (IBM Corp, 2021). We used the PROC TRAJ package in SAS software for group-based trajectory modeling. Figure 2 summarizes the data analysis plan.

### Identifying Heterogeneity in Latent Trajectories (Research Question 1)

In a first step, we used a person-oriented group-based method to identify the trajectories for felt belongingness, social support, self-esteem, and life satisfaction separately (Jones et al., 2001; Jung & Wickrama, 2008). Using finite mixtures of suitably defined probability distributions (Nagin, 1999), the group-based approach for multinomial modelling of trajectories is a method that identifies distinctive clusters of individual trajectories within the population. This can then be used for profiling the characteristics of individuals within these clusters. In line with existing statistical practices (Wollast et al., 2023), we estimated multiple latent classes (i.e., trajectories) for each variable independently. Once we determined the optimal number of trajectories, we assessed whether the trajectories were characterized by a constant, linear, quadratic, or cubic function. It is important to note that the group-based trajectory modeling approach assumes that within-group variance is zero, meaning that all individuals within a trajectory group follow the same estimated trajectory without individual deviations.

To model the trajectory data, we used the CNORM (Censored Normal) distribution, which allows for the underlying latent variable to be normally distributed. This distribution permits values outside the observed scale bounds, providing flexibility in capturing the potential for extreme values, even though such values are not directly observed in the data.

A key challenge in finite mixture modeling is determining the optimal number of trajectory groups and their shape for the best-fitting model. Following the recommendations of D’Unger et al. (1998), we selected the optimal model based on the Bayesian Information Criterion (BIC), where the model with the least negative BIC value (i.e., the BIC value closest to zero) indicates the best fit. More precisely, we relied on the conventional BIC (which accounts for sample size and model complexity) and the Schwarz BIC (which applies a stronger penalty for model complexity) to determine the best-fitting model. Additionally, we reported the log-likelihood for each model, though this measure should not be used as a standalone fit index for model comparison. Ultimately, we did not report the AIC, as it is often problematic for Latent Class Growth Analysis (LCGA) and less precise than the BIC (Nylund-Gibson et al., 2007).

Taking these indices into account, we examined the magnitude of the estimates and the significance of each slope to determine the best combination. Based on the selected model, these procedures assign people into trajectory groups based on the maximum posterior probability. We expected that the trajectories would differ in starting points of each well-being variable.

### Co-Occurrence Using Joint Latent Trajectories (Research Question 2)

Step 2 involved examining the probability of belonging in a trajectory group on one variable based on belonging to a specific trajectory group on another variable. Because we included four well-being variables, we conducted six paired analyses. We expected that the four trajectories would be interconnected, for example, that people following a low or high self-esteem trajectory would also follow a low or high life satisfaction trajectory, respectively.

### Identifying Multitrajectories

After assigning individuals into different trajectory groups (step 1) and confirming that these different trajectory groups were strongly and positively associated with each other (step 2), we identified groups of multiple trajectories. While step 1 identified clusters of participants for each well-being indicator separately, the multitrajectory approach identified groups of individuals that followed similar patterns across the four well-being variables considered simultaneously. We expected that when felt belongingness, social support, self-esteem, and life satisfaction were considered jointly in the same multitrajectory model, trajectories would follow the same pattern as observed in our first step. Multitrajectory is a generalization of group-based trajectory modeling which allowed us to take full advantage of the information available in multivariate longitudinal data for tracking the course of well-being (Nagin et al., 2018). While group-based trajectory modeling is designed to identify clusters of individuals who are following similar trajectories of a single indicator of interest (steps 1 and 2), multitrajectory modeling identifies latent clusters of individuals following similar trajectories across multiple indicators (e.g., felt belongingness, social support, self-esteem, and life satisfaction) of an outcome of interest (e.g., well-being). These multitrajectory group membership designations enabled us to create groups with different levels of overall well-being. Consequently, the percentage of participants belonging to specific multitrajectory groups of well-being is the same for each well-being variable, whereas this percentage varies between variables in the first group-based trajectory modeling approach (steps 1 and 2).

### Demographic Characteristics Associated with Trajectories (Research Question 3)

Finally, in step 3, we assessed the predictive roles of education, age, gender, and ethnicity in affecting the likelihood of membership in a given trajectory. Similar to other person-oriented methods (Osborne & Sibley, 2017), we employed logistic regression as a means to predict trajectory group membership. Because education was only available as a predictor for those who participated in Wave 1 (*N* = 4,845 participants), we estimated models with and without education as a predictor. As a general rule, we discussed the results using all predictors, but note when results in the models omitting education differ.

## Results

### Identification of Well-Being Trajectories

To address the first research question, we identified the number and the shape of trajectories for felt belongingness, social support, self-esteem, and life satisfaction, separately, from wave 2 (2010) to wave 13 (2021). Wave 1 was excluded to guarantee that our examination of the influences of sociodemographic variables and potential psychological constructs incorporates predictors assessed before the trajectory initiation. This common strategic approach aimed to establish preliminary evidence for causal inferences while mitigating overlaps (Nagin et al., 2018; Wollast et al., 2023). Table 1 reports *BIC* scores and related indices for models with varying numbers of groups (i.e., trajectories) and trajectory shapes within each group.

**Table 1.** Information Criterion for Selection of Models (Step 1).

Trajectory	Model	*K*	Order	BIC 1	BIC 2	LL
Trajectories of felt belongingness	1	2	3, 3	−312,095	−312,087	−312,034
	2	3	3, 3, 3	−296,812	−296,800	−296,720
	3	4	3, 3, 3, 3	−290,690	−290,673	−290,566
	4	4	1, 0, 2, 1	−290,651	−290,641	−290,577
	5	5	3, 3, 3, 3, 3	−287,863	−287,843	−287,709
	**6**	**5**	**1, 1, 1, 1, 1**	−**287,822**	−**287,810**	−287,729
Trajectories of social support	1	2	3, 3	−328,121	−328,113	−328,059
	2	3	3, 3, 3	−309,618	−309,606	−309,525
	3	4	3, 3, 3, 3	−303,687	−303,671	−303,564
	4	4	1, 1, 2, 0	−303,649	−303,639	−303,575
	5	5	3, 3, 3, 3, 3	−301,688	−301,667	−301,533
	**6**	**5**	**1, 1, 1, 2, 0**	−**301,634**	−**301,622**	−301,541
Trajectories of self-esteem	1	2	3, 3	−342,166	−342,157	−342,114
	2	3	3, 3, 3	−323,103	−323,091	−323,025
	3	4	3, 3, 3, 3	−314,693	−314,676	−314,569
	4	4	3, 3, 3, 1	−314,689	−314,674	−314,577
	5	5	3, 3, 3, 3, 3	−310,238	−310,217	−310,083
	**6**	**5**	**2, 3, 3, 3, 1**	−**310,226**	−**310,207**	−310,090
Trajectories of life satisfaction	1	2	3, 3	−340,472	−340,464	−340,411
	2	3	3, 3, 3	−322,706	−322,694	−322,614
	3	4	3, 3, 3, 3	−313,296	−313,280	−313,172
	4	4	2, 3, 3, 3	−313,295	−313,279	−313,177
	5	5	3, 3, 3, 3, 3	−308,890	−308,869	−308,736
	**6**	**5**	**2, 3, 3, 3, 3**	−**308,885**	−**308,865**	−308,737
Multitrajectories of well-being when all four indicators are taken simultaneously	1	FB	3, 3, 3, 3, 3	−1,251,606	−1,251,472	−1,251,000
		SS	3, 3, 3, 3, 3			
		SE	3, 3, 3, 3, 3			
		LS	3, 3, 3, 3, 3			
	**2**	**FB**	**1, 1, 1, 1, 1**	−**1,251,463**	−**1,251,367**	−1,251,029
		**SS**	**1, 1, 0, 2, 0**			
		**SE**	**2, 3, 3, 3, 1**			
		**LS**	**2, 3, 3, 3, 3**			

*Note. K* = Number of groups (trajectories). BIC 1 = Schwarz BIC. BIC 2 = Conventional BIC. LL = log likelihood. FB = Felt Belongingness. SS = Social Support. SE = Self-Esteem. LS = Life Satisfaction. The order indicates whether the trajectory was fit with a constant (0), linear (1), quadratic (2), or cubic (3) function according to model fit statistics. For each *k* level, we tested all model combinations possible including all different polynomial functions. For clarity, we only report the most relevant models (baseline model and best model for four and five trajectory groups). Lines in bold represent the best fit according to the model fit statistics.

**Figure 3.**
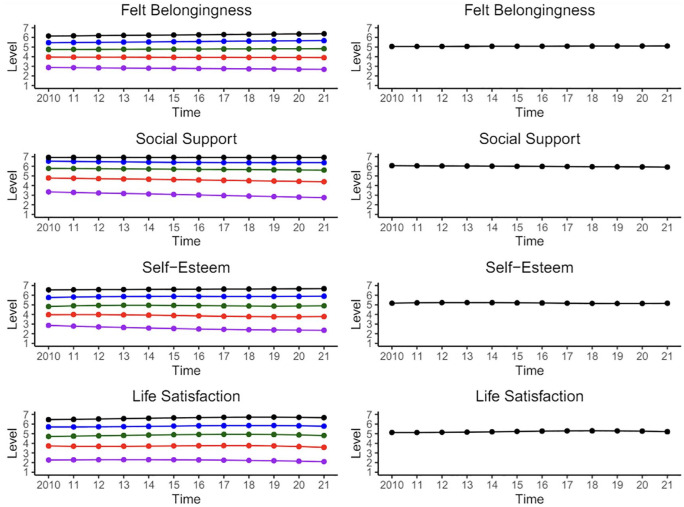
Pattern of trajectories for the four well-being variables as a function of trajectory groups (on the left) and for only one latent trajectory (on the right). The *x*-axis (horizontal line) represents the twelve waves of data collection, whereas the *y*-axis (vertical line) represents the seven levels of the well-being response scale. Model fit statistics can be found in Tables 1–5.

Based on the BIC criterion, a six-group model was identified as the best fitting model for all well-being indicators separately. However, because models including six or more trajectory groups generated trajectories containing a low number of participants (i.e., less than 1% of the sample), we selected as the best model the five-group model, which contained sufficient participants in each latent cluster to provide appropriate power to conduct subsequent analyses.^1^ The parameters of the chosen models are presented in Tables 2–5. Figure 3 depicts the patterns of the trajectories for each well-being variable, as well as the overall latent trend line for the sample. The five trajectory groups show substantial heterogeneity in well-being that is not represented in the overall latent trend.

**Table 2.** Parameters for the Selected Group-Based Trajectory Model of Felt Belongingness.

Trajectory groups	Parameter	Estimate	Standard error	*T* for H0	Prob > |*T*|
Very high (*N* = 12.4%)	Intercept	6.12667	0.01601	382.638	0.0000
	**Linear**	**2.55884**	**0.15305**	**16.719**	**0.0000**
High (*N* = 37.1%)	Intercept	5.41780	0.01118	484.535	0.0000
	**Linear**	**2.00890**	**0.09326**	**21.540**	**0.0000**
Moderate-high (*N* = 31.8%)	Intercept	4.72613	0.01238	381.700	0.0000
	**Linear**	**0.80682**	**0.10575**	**7.629**	**0.0000**
Moderate (*N* = 15.2%)	Intercept	3.97109	0.01666	238.323	0.0000
	**Linear**	−**0.52886**	**0.15659**	−**3.377**	**0.0007**
Low (*N* = 3.5%)	Intercept	2.91523	0.03326	87.654	0.0000
	**Linear**	−**1.82627**	**0.31835**	−**5.737**	**0.0000**
	Sigma	0.66840	0.00107	625.714	0.0000

*Note*. Sigma represents the parameters associated with the estimated standard deviation of the residuals.

Lines in bold represent significant estimates derived from the most complex model that still improved fit.

**Table 3.** Parameters for the Selected Group-Based Trajectory Model of Social Support.

Trajectory groups	Parameter	Estimate	Standard error	*T* for H0	Prob > |*T*|
Very high (*N* = 20.3%)	**Intercept**	**7.79415**	**0.01424**	**547.507**	**0.0000**
High (*N* = 34.6%)	Intercept	6.90374	0.03429	201.361	0.0000
	Linear	−6.44356	0.75501	−8.534	0.0000
	**Quadratic**	**28.45677**	**4.40888**	**6.454**	**0.0000**
Moderate-high (*N* = 31.2%)	Intercept	5.84697	0.01977	295.766	0.0000
	**Linear**	−**1.71132**	**0.14133**	−**12.109**	**0.0000**
Moderate (*N* = 11.0%)	Intercept	4.86399	0.02666	182.453	0.0000
	**Linear**	−**3.59686**	**0.24124**	−**14.910**	**0.0000**
Low (*N* = 2.9%)	Intercept	3.45024	0.04612	74.817	0.0000
	**Linear**	−**5.46803**	**0.44388**	−**12.319**	**0.0000**
	Sigma	0.87445	0.00170	514.359	0.0000

*Note*. Sigma represents the parameters associated with the estimated standard deviation of the residuals.

Lines in bold represent significant estimates derived from the most complex model that still improved fit.

**Table 4.** Parameters for the Selected Group-Based Trajectory Model of Self-Esteem.

Trajectory groups	Parameter	Estimate	Standard error	*T* for H0	Prob > |*T*|
Very high (*N* = 10.5%)	Intercept	6.69651	0.01985	337.311	0.0000
	**Linear**	**1.91109**	**0.**1992**5**	**9.592**	**0.0000**
High (*N* = 38.6%)	Intercept	5.60367	0.04165	134.532	0.0000
	Linear	**11.05659**	**1.80192**	**6.136**	**0.0000**
	Quadratic	−**135.93874**	**23.86761**	−**5.696**	**0.0000**
	**Cubic**	**533.02406**	**97.74059**	**5.453**	**0.0000**
Moderate-high (*N* = 29.5%)	Intercept	4.58136	0.05159	88.808	0.0000
	Linear	16.69914	2.22195	7.516	0.0000
	Quadratic	−230.85681	29.43077	−7.844	0.0000
	**Cubic**	**931.69848**	**120.62955**	**7.724**	**0.0000**
Moderate (*N* = 16.6%)	Intercept	3.87728	0.07221	53.697	0.0000
	Linear	7.91602	3.07685	2.573	0.0101
	Quadratic	−162.83377	40.38747	−4.032	0.0001
	**Cubic**	**739.15723**	**164.40778**	**4.496**	**0.0000**
Low (*N* = 4.8%)	Intercept	3.04148	0.06353	47.873	0.0000
	Linear	−9.65466	1.57202	−6.142	0.0000
	**Quadratic**	**33.10949**	**9.19568**	**3.601**	**0.0003**
	Sigma	0.73601	0.00120	611.833	0.0000

*Note*. Sigma represents the parameters associated with the estimated standard deviation of the residuals.

Lines in bold represent significant estimates derived from the most complex model that still improved fit.

**Table 5.** Parameters for the Selected Group-Based Trajectory Model of Life Satisfaction.

Trajectory groups	Parameter	Estimate	Standard error	*T* for H0	Prob > |*T*|
Very high (*N* = 10.1%)	Intercept	6.61569	0.08788	75.282	0.0000
	Linear	−3.59000	3.87577	−0.926	0.3543
	Quadratic	182.42739	51.77648	3.523	0.0004
	**Cubic**	−**1044.43808**	**213.36991**	−**4.895**	**0.0000**
High (*N* = 45.1%)	Intercept	5.78815	0.04029	143.651	0.0000
	Linear	−5.97094	1.73092	−3.450	0.0006
	Quadratic	140.51849	22.79931	6.163	0.0000
	**Cubic**	−**724.42350**	**93.00956**	−**7.789**	**0.0000**
Moderate-high (*N* = 29.4%)	Intercept	4.68414	0.05288	88.584	0.0000
	Linear	0.22544	2.26739	0.099	0.9208
	Quadratic	78.40577	29.96716	2.616	0.0089
	**Cubic**	−**560.18244**	**122.62833**	−**4.568**	**0.0000**
Moderate (*N* = 12.1%)	Intercept	3.89202	0.08182	47.567	0.0000
	Linear	−12.35393	3.56372	−3.467	0.0005
	Quadratic	223.11915	47.24336	4.723	0.0000
	**Cubic**	−**1130.94453**	**193.73279**	−**5.838**	**0.0000**
Low (*N* = 3.3%)	Intercept	2.19735	0.07403	29.681	0.0000
	Linear	3.46371	1.90025	1.823	0.0683
	**Quadratic**	−**33.96138**	**11.31482**	−**3.001**	**0.0027**
	Sigma	0.75176	0.00125	603.081	0.0000

*Note*. Sigma represents the parameters associated with the estimated standard deviation of the residuals.

Lines in bold represent significant estimates derived from the most complex model that still improved fit.

In line with our expectations, the main differences between our trajectories reflected initial levels of well-being (see Figure 3). Across all four indicators of well-being, one trajectory signified low well-being (purple), another signified moderate well-being (red), a third signified moderate-high well-being (green), a fourth signified high well-being (blue), and the fifth signified very high well-being (black).^2^

The very high well-being trajectory group comprised of 12.4% (felt belongingness), 20.3% (social support), 10.5% (self-esteem), and 10.1% (life satisfaction) of the sample. Among this group, social support displayed a constant trajectory. Felt belongingness and self-esteem can both be characterized by a small, but statistically significant linear upward trajectory. By contrast, life satisfaction displayed a negative cubic function. In other words, life satisfaction displayed a positive trajectory until the middle of the panel, and then it fluctuated within a narrow band for the rest of the observed period.

The high trajectory characterized a greater percentage of participants (37.1% for felt belongingness, 34.6% for social support, 38.6% for self-esteem, and 45.1% for life satisfaction). The high felt belongingness trajectory was characterized by small but accelerating positive change. In contrast, the high social support trajectory was characterized by a positive quadratic function, indicating a concave downward pattern over the observed period. By contrast, self-esteem demonstrated a positive cubic function, indicating a pattern of fluctuation within a narrow range over the observed period. The positive cubic nature suggests an upward curvature, typically associated with a convex shape. In parallel, life satisfaction displayed a negative cubic function, implying a similar pattern of fluctuation within a narrow band over the observed period. The negative cubic nature indicates a downward curvature, typically associated with a concave shape.

The moderate-high trajectory was also characterized by a greater percentage of participants (31.8% for felt belongingness, 31.2% for social support, 29.5% for self-esteem, and 29.4% for life satisfaction). Felt belongingness and self-esteem can both be described by a small but statistically significant linear trajectory, with a positive estimation for the former and a negative estimation for the latter. Again, self-esteem and life satisfaction displayed a positive and negative cubic function, respectively.

The moderate trajectories contained a fair number of participants as well (15.2% for felt belongingness, 11.0% for social support, 16.6% for self-esteem, and 12.1% for life satisfaction). Felt belongingness and social support saw a small, but statistically significant decline throughout the period of observation. Just like the moderate trajectories, the moderate trajectories of self-esteem and life satisfaction displayed a positive and negative cubic function, respectively.

A similar pattern emerged for individuals demonstrating a low level of well-being. These individuals were not highly prevalent, comprising just 3.5% (felt belongingness), 2.9% (social support), 4.8% (self-esteem), and 3.3% (life satisfaction) of the sample. Marked linear declines were observed in felt belongingness and social support. Again, the low trajectories of self-esteem and life satisfaction displayed a positive and negative cubic function, respectively.

In summary, our findings revealed that well-being trajectories are largely static. However, an asynchronous divergent pattern emerged across well-being indicators. In contrast to the highest well-being trajectories, the lowest well-being trajectories demonstrated an overall decline, diverging further from higher well-being trajectory groups. Interestingly, this decline was more pronounced during the COVID-19 pandemic.

### Conditional Probabilities of Co-Occurrence

Next, we examined the co-occurrence of the trajectory groups of the four well-being variables. Table 6 reports the conditional probabilities of belonging to a specific trajectory group conditional to one of the three others. In line with expectations, the four sets of well-being trajectories strongly coincided with each other, suggesting that these four well-being indicators tend to follow a comparable course over time. Specifically, the large diagonal elements of the probability matrixes (in bold in Table 6) indicate that there is considerable overlap between these trajectories.

**Table 6.** Co-Occurrence of the Four Well-Being Variables (Step 2).

Group	Low	Moderate	Moderate-high	High	Very high
Probability of felt belongingness groups conditional on social support groups					
Very high felt belongingness	0.1	0.1	0.1	6.1	**48.3**
High felt belongingness	1.3	2.1	19.3	**61.1**	39.8
Moderate-high felt belongingness	5.1	27.9	**57.0**	27.7	10.1
Moderate felt belongingness	36.8	**57.2**	21.8	4.8	1.6
Low felt belongingness	**56.7**	12.7	1.8	0.3	0.2
Probability of felt belongingness groups conditional on self-esteem groups					
Very high felt belongingness	0.0	0.3	1.0	9.3	**67.3**
High felt belongingness	2.2	5.5	24.1	**67.4**	26.6
Moderate-high felt belongingness	9.3	34.5	**59.8**	20.3	4.5
Moderate felt belongingness	44.8	**52.0**	13.9	2.8	1.5
Low felt belongingness	**43.7**	7.7	1.2	0.2	0.1
Probability of felt belongingness groups conditional on life satisfaction groups					
Very high felt belongingness	0.5	0.2	1.6	12.2	**56.5**
High felt belongingness	3.0	6.4	22.8	**59.0**	32.7
Moderate-high felt belongingness	11.0	30.5	**53.7**	24.9	8.7
Moderate felt belongingness	36.6	**52.0**	20.2	3.6	2.1
Low felt belongingness	**48.9**	10.9	1.7	0.3	0.0
Probability of social support groups conditional on self-esteem groups					
Very high social support	4.1	7.4	11.5	23.0	**64.6**
High social support	14.1	21.2	31.9	**48.9**	29.1
Moderate-high social support	28.9	38.5	**42.0**	24.5	4.5
Moderate social support	30.3	**26.4**	12.9	3.0	1.5
Low social support	**22.6**	6.5	1.7	0.6	0.3
Probability of social support groups conditional on life satisfaction groups					
Very high social support	3.1	3.7	8.0	23.4	**67.8**
High social support	8.7	12.9	27.3	**50.7**	27.1
Moderate-high social support	19.9	38.2	**48.5**	22.9	3.8
Moderate social support	32.7	**35.7**	14.5	2.7	1.1
Low social support	**35.6**	9.5	1.7	0.3	0.2
Probability of self-esteem groups conditional on life satisfaction groups					
Very high self-esteem	0.0	0.2	0.7	8.2	**63.5**
High self-esteem	1.2	2.6	19.4	**65.5**	30.2
Moderate-high self-esteem	4.8	23.7	**54.6**	22.7	5.3
Moderate self-esteem	28.2	**58.3**	23.2	3.3	1.0
Low self-esteem	**65.8**	15.2	2.1	0.3	0.0

For instance, individuals who follow the trajectory with the lowest level of belongingness also follow the lowest level trajectories of social support (56.7%), self-esteem (43.7%), and life satisfaction (48.9%). A similar pattern emerged for the highest level of well-being with people who follow the highest level of belongingness also following the highest level of social support (48.3%), self-esteem (67.3%), and life satisfaction (56.5%). In sum, a strong overlap was observed across the four well-being variables.

We do note two exceptions, however. Those who felt the highest social support often felt the highest self-esteem (64.6%) and life satisfaction (67.8%). However, those who experienced the lowest social support trajectory did not necessarily experience the lowest trajectories of self-esteem (22.6%) or life satisfaction (35.6%). In fact, it was more common for those with the lowest trajectories of life satisfaction and self-esteem to display moderately low trajectories of social support (39.8% and 42.0%, respectively). Within-wave, the average correlation of social support with self-esteem (*r*_range_ = .31−.42) and with life satisfaction (*r*_range_ = .41−.48) is weaker than its correlation with felt belongingness (*r*_range_ = .41−.57), which may be why trajectory group membership overlaps less between social support and other indicators.

### Multitrajectories of Well-Being

After identifying individual trajectories for each well-being indicator, we conducted a multitrajectory analysis to simultaneously examine the joint evolution of felt belongingness, social support, self-esteem, and life satisfaction (Nagin et al., 2018). This analysis yielded five distinct latent classes: low (4.1%), moderate (14.8%), moderate-high (29.6%), high (36.6%), and very high (14.9%) levels of well-being (see Table 1 for model selection details). The trajectory patterns, along with their 95% confidence intervals, are provided in the online Supplemental Material.

Notably, the best-fitting model revealed that the trajectories of the four well-being indicators, when modeled jointly, closely mirrored the patterns observed when each indicator was analyzed separately. The only exception was the moderate trajectory of felt belongingness, which remained constant rather than showing the linear trend identified in the single-trajectory analysis (step 1). This consistency across models reinforces the idea that these four well-being indicators are interrelated and capture distinct yet converging aspects of a broader well-being construct.

These findings suggest that well-being develops in a similar manner across its sub-dimensions. The strong alignment between single- and multitrajectory models highlights the robustness of the identified patterns, providing further evidence that these well-being dimensions fluctuate together over time rather than evolving independently.

### Associations Between Trajectories and Sociodemographic Factors

Our third research question concerns whether there are key demographic differences in various indicators of well-being. To this end, we estimated a series of logistic regression models predicting trajectory group membership. We entered linear, quadratic, and cubic terms for age, gender (men as the reference category), education, and ethnicity (NZ Europeans as the reference category) as predictors. Since coefficients are not as intuitive with logistic regression models as they are with linear regression, we employed the use of predicted probability plots derived from these models in the main text. However, the results of the models, with all combinations of comparisons, can be found in Tables S3–S6 of the Supplemental Material (Tables S7–S10 without education).

#### Age

**Figure 4.**
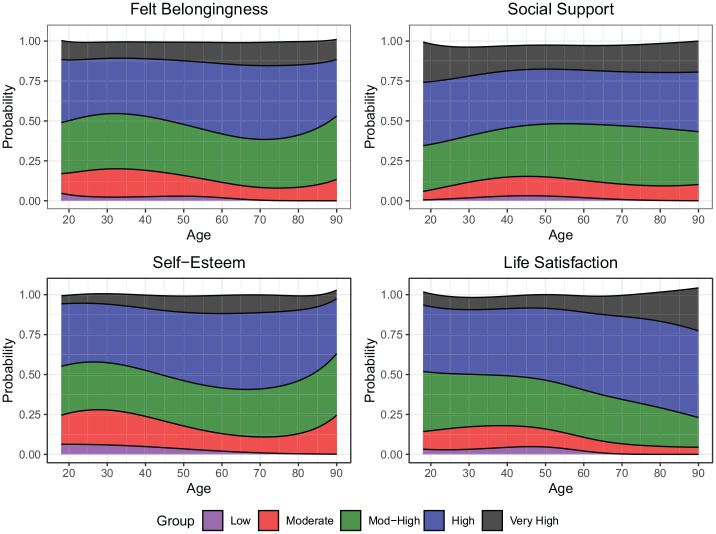
Likelihood of trajectory membership (*y*-axis) as a function of age (*x*-axis). Predicted probabilities derived from logistic regression models from Tables S3–S6 in the Supplemental Materials.

First, we examined the association between age and trajectory group membership. Across well-being indicators (see Figure 4 for predicted probabilities of trajectory group membership), the linear, quadratic, and cubic terms showed joint statistical significance in 17/20 models that include education (*p*s ≤ .019) and 19/20 models omitting education (*p*s < .001). However, the patterns of trajectory membership varied somewhat by indicator.

Generally, older respondents (e.g., 60+ years) have a very low likelihood of being in the lowest well-being trajectory. Out of any age group, the youngest adults (e.g., 20–40 years) are most likely to belong to the low well-being trajectory across indicators (except for social support which is highest among young adults), though middle-aged adults (e.g., 40–60 years) are also well-represented in the low life satisfaction and low social support trajectory groups. Membership in the highest well-being trajectory group is either stable across age (e.g., felt belongingness, social support, self-esteem) or becomes more likely with age (e.g., life satisfaction). Membership of the moderate and moderate-high trajectories peaks in middle age for social support and younger adulthood for felt-belongingness, self-esteem, and life satisfaction. People in retirement age (e.g., 60+ years) are particularly well represented in the higher well-being trajectories (with the exception of social support). However, the oldest old (e.g., 80+ years) are more likely to belong to lower belongingness and self-esteem trajectories compared to those in earlier retirement years.

#### Gender

**Figure 5.**
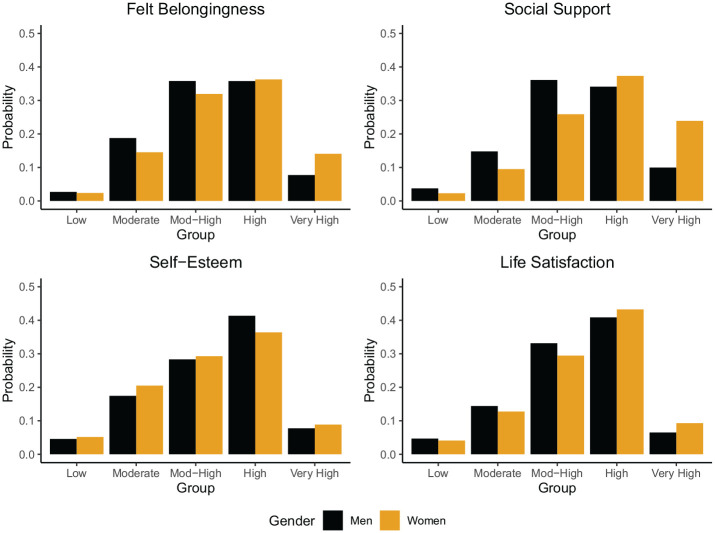
Likelihood of trajectory membership (*y*-axis) as a function of gender (grouped on *x*-axis). Predicted probabilities derived from logistic regression models from Tables S3–S6 in the Supplemental Materials.

Across well-being indicators, gender shows a significant association with the likelihood of group membership in 12/20 models that include education and 18/20 models that exclude education. The predicted probabilities of trajectory group membership are shown in Figure 5. Women are less likely than men to belong to the lowest felt-belongingness, social support, and life satisfaction trajectory groups, and more likely than men to belong to the highest felt-belongingness, social support, and life satisfaction trajectory groups. However, for self-esteem, the pattern is partly reversed. Women are significantly more likely than men to belong to the moderate and moderate-high self-esteem trajectory groups, while men are more likely to belong to the high trajectory group. There are no significant gender differences in membership of the low and very high trajectory groups for self-esteem.

#### Education

**Figure 6.**
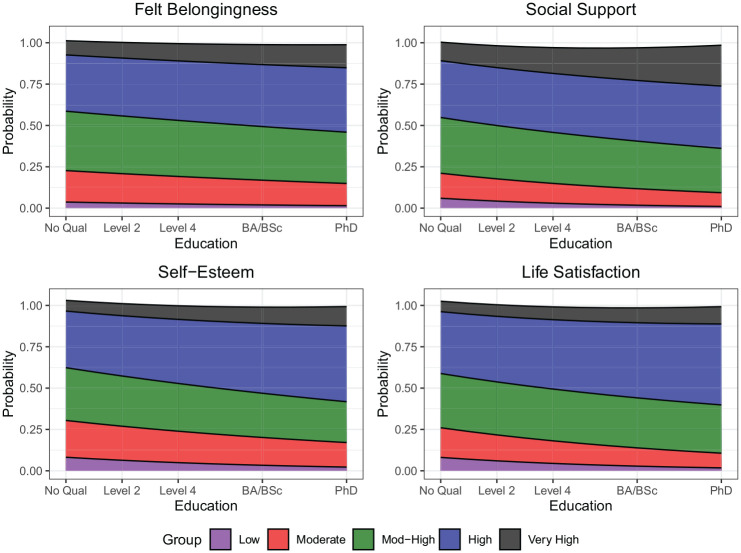
Likelihood of trajectory membership (*y*-axis) as a function of education (*x*-axis). Predicted probabilities derived from logistic regression models from Tables S3–S6 in the Supplemental Materials.

Across well-being indicators, education shows a significant association with the likelihood of trajectory group membership in 16/20 models. The predicted probabilities of trajectory group membership are shown in Figure 6. Across well-being indicators, individuals with lower levels of educational attainment are more likely to belong to the lowest trajectory groups. Conversely, individuals with higher levels of educational attainment are more likely to belong to the highest trajectory groups.

#### Ethnicity

Across well-being indicators, the ethnic group terms were jointly statistically significant in 7/20 models that included education and 18/20 models that excluded education. This difference could reflect higher statistical power in models excluding education. However, it is also possible that differences between ethnic groups are partially accounted for by education.

**Figure 7.**
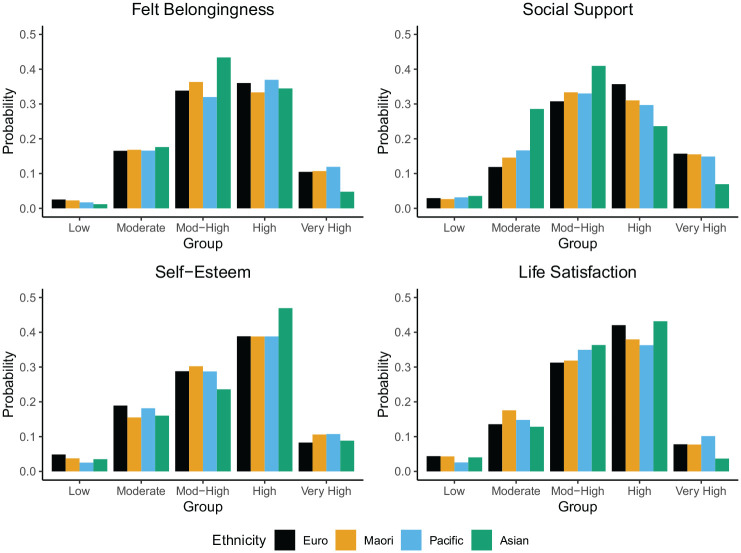
Likelihood of trajectory membership (*y*-axis) as a function of ethnicity (grouped on *x*-axis). Predicted probabilities derived from logistic regression models from Tables S3–S6 in the Supplemental Materials.

The results were complex and varied across well-being indicators, ethnic groups, and models. Therefore, we report key findings here (see Figure 7 for a summary) and provide the full results in the Supplemental Materials. For felt belongingness and social support, Māori New Zealanders and Asian New Zealanders are more likely, relative to European New Zealanders, to belong to lower trajectory groups and less likely to belong to higher trajectory groups. Pacific New Zealanders are similar to European New Zealanders across all models of felt belongingness and social support. For self-esteem, Māori, Asian, and Pacific New Zealanders are more likely than European New Zealanders to belong to high self-esteem trajectory groups and less likely to belong to low self-esteem trajectory groups. In contrast, for life satisfaction, non-white New Zealanders are generally more likely, relative to European New Zealanders, to belong to lower trajectory groups and less likely to belong to higher trajectory groups.

## Discussion

In this article, we examined heterogeneity in trajectories of well-being across multiple indicators using a large, nationally representative longitudinal probability sample of New Zealanders over a period of 13 years using 13 waves of data collection from 2009 to 2021. First, in line with our hypotheses, we identified clusters of individuals following persistently low, moderate, moderate-high, high, and very high trajectories of felt belongingness, social support, self-esteem, and life satisfaction, with differences and similarities in their stability over time. Second, we found a strong overlap between trajectories across well-being indicators. Third, we observed several education, age, gender, and ethnic differences characterizing these trajectories.

Our analyses reveal substantial heterogeneity in trajectories of well-being, mostly represented by quantitative differences in initial well-being levels. People who have high (low) levels of well-being at the beginning of observation remain at higher (lower) levels of well-being throughout observation. Most people’s well-being can be characterized as moderate or high, and few people display low levels of well-being. Specifically, we identified one small group with low well-being (2.9%–4.8% of the sample), and four further groups with moderate well-being (11.0%–16.6%), moderate-high well-being (29.4%–31.8%), high well-being (34.6%–45.1%), and very high well-being (10.1%–20.3%). Accordingly, our results paint a largely positive picture regarding well-being levels in the New Zealand population.

Across most, but not all pairs of indicators, we also found that membership of one high (low) well-being trajectory is strongly predictive of membership of another high (low) well-being trajectory. This indicates that different components of well-being may go hand in hand. For example, high social support trajectories accompany high trajectories in self-esteem and life satisfaction. Our multitrajectory analysis further confirmed this interdependence, revealing that well-being develops in a similar manner across its sub-dimensions. The strong alignment between single- and multitrajectory models suggests that these well-being indicators fluctuate together over time rather than evolving independently. Our findings therefore extend prior research that has reported positive correlations between different indicators of well-being (e.g., Diener & Diener, 1995; Howard et al., 2022). Future research may seek to identify the reasons for these links. Different components of well-being may co-occur or be causally linked, for example, perhaps greater social support may increase one’s felt belongingness and life satisfaction.

However, we also find important heterogeneity in trajectories beyond starting values. Most trajectories are characterized by stability or fluctuation within an extremely narrow band. This suggests, out of our typology, that different well-being trajectories are largely static. There is one exception, however, that signals partial divergence (i.e., an asynchronous divergent pattern). Across indicators, people who have already begun with the lowest well-being experience, decline, sometimes accelerating, in their well-being. By implication, their levels of well-being become increasingly distinct, and in a negative manner, from the other trajectory groups. Thus, although we observed the greatest heterogeneity in levels of well-being, we also identified key differences between groups in well-being trajectories over time, demonstrating the value of using longitudinal data to study changes in well-being. While future research will need to delve more carefully into the low well-being group, this research suggests that people suffering from chronically low well-being might be prone to downward spirals over time. With regard to the practical implications of our research, these results suggest that targeted interventions to improve well-being could be particularly beneficial for the low-well-being group, especially if the intervention is implemented early to prevent declines over time.

In general, our findings on the demographic predictors of long-term well-being trajectories are in line with cross-sectional studies of between-person differences in well-being (e.g., Frei & Axhausen, 2007; McLaren et al., 2001; Painter, 2013; Wollast et al., 2025). For instance, we replicate findings that higher education is associated with higher well-being trajectories. In line with a number of cross-sectional studies, we found that women experience higher levels of felt belongingness, social support, and life satisfaction than men. However, for self-esteem, the pattern is partly reversed. Women are significantly more likely than men to be in the moderate self-esteem trajectory groups, while men are more likely to be in the high trajectory group. We found that the effects of age are complex and contingent on the indicator. This finding suggests that the inconsistent results found in other studies are perhaps due to the use of diverging indicators of well-being. The effects of ethnicity were also complex and differed across well-being indicators and ethnic groups. For example, we found that Māori, Asian, and Pacific New Zealanders were more likely than European New Zealanders to be in high self-esteem trajectory groups, but less likely to be in high social support trajectory groups. It is possible that ongoing prejudice and discrimination toward minoritized ethnic groups may have detrimental consequences for specific aspects of well-being, such as feelings of loneliness and social exclusion (Benner et al., 2022; Benner & Wang, 2017; Vines et al., 2017), whereas self-esteem may benefit from an early increase in adolescence sometimes observed among minoritized individuals (Erol & Orth, 2011; Orth et al., 2010; Shaw et al., 2010). Exploring these and other potential reasons for the divergent trajectories across ethnic groups and well-being indicators presents a fruitful avenue for future research. Nonetheless, our findings overall suggest that longitudinal methods closely track and mirror the cross-sectional differences identified in prior research.

Our study has several key limitations. First, we used short-form measures of each indicator of well-being, and in particular, felt belongingness suffered psychometric consequences. Shortening these measures was pragmatic given the need to include a variety of indicators in the NZAVS. Importantly, the majority of short-form scales employed by the NZAVS demonstrate adequate internal reliability compared to their original full-form versions and issues with specific scales are discussed (Sibley et al., 2024). Furthermore, it has been well established that the quality of measurement plays a significant role in identifying trajectory classes (Bauer, 2007; Cole et al., 2017). Therefore, the use of short-form scales and the poor composite reliability of felt belongingness should be considered as potential limitations that could have influenced the model outcomes. Second, we focus on four indicators of well-being (felt belongingness, social support, self-esteem, and life satisfaction), but well-being is a broad construct that could be measured with various additional indicators, such as affective dimensions. Third, due to missing data, we did not examine other demographic factors (e.g., income, religion) or life experiences that might affect trajectories of well-being. We encourage future research to expand on our work by examining the role of these factors over time. Fourth, while a high-quality sample, our study is restricted to New Zealand adults, meaning inferences are not necessarily generalizable to children or adolescents or people beyond New Zealand without further research. Finally, our use of annual measurements leaves open the possibility that more fluctuation occurs at shorter time scales.

Our study also has a number of important strengths. First, we use a large, nationally representative probability sample of New Zealanders, meaning our analyses can make plausible inferences about the New Zealand population. This also means that our sample is more varied than most student or online convenience samples. We further observe respondents over an incredibly long time scale, enabling us to examine how well-being changes across large sections of respondents’ lives. Our large sample size and sophisticated modeling approach allow for a nuanced examination of heterogeneity in well-being across time, which cannot be captured by modeling techniques that focus instead on overall trends. Furthermore, our ability to use multiple indicators of well-being means that our results are not an artifact of one indicator or another.

In conclusion, this study represents an important step in understanding how well-being operates over time within both people and larger populations. By applying a group-based trajectory modeling approach to large-scale longitudinal data, we uncovered meaningful heterogeneity in well-being trajectories. While most people maintain moderate to high levels of well-being, those with lower initial well-being are more likely to experience further declines, highlighting the need for early, targeted interventions to prevent downward spirals. The strong overlap across indicators like social support, self-esteem, and life satisfaction suggests that improvements in one area could positively influence others, evidence of the interconnected nature of well-being. Ultimately, by identifying those at greatest risk and understanding the sociodemographic and psychological factors influencing their trajectories, we can better design preventive mental health applications and resources to improve well-being across diverse populations.

## Supplemental Material

sj-docx-1-psp-10.1177_01461672251331654 – Supplemental material for Modeling Heterogeneity in the Long-Term Trajectories of Individuals’ Well-Being Supplemental material, sj-docx-1-psp-10.1177_01461672251331654 for Modeling Heterogeneity in the Long-Term Trajectories of Individuals’ Well-Being by Robin Wollast, Joseph B. Phillips, Chloe Bracegirdle, Olivia Spiegler, Chris G. Sibley, Éric Lacourse and Nikhil K. Sengupta in Personality and Social Psychology Bulletin

sj-zip-2-psp-10.1177_01461672251331654 – Supplemental material for Modeling Heterogeneity in the Long-Term Trajectories of Individuals’ Well-Being Supplemental material, sj-zip-2-psp-10.1177_01461672251331654 for Modeling Heterogeneity in the Long-Term Trajectories of Individuals’ Well-Being by Robin Wollast, Joseph B. Phillips, Chloe Bracegirdle, Olivia Spiegler, Chris G. Sibley, Éric Lacourse and Nikhil K. Sengupta in Personality and Social Psychology Bulletin

## Notes

1. The smallest cluster in the five-group model comprises 2.9% (representing the low trajectory group for social contact), equivalent to 1,310 participants. This provides sufficient statistical power to detect the significance of slopes within trajectory classes and allows us to conduct subsequent analyses (Frankfurt et al., 2016).

2. As a manipulation check, we report the average posterior probability of assignment (APPA; Asparouhov & Muthén, 2014) estimating the probability of belonging to each trajectory group conditional on longitudinal responses (see Table S2). The APPA is calculated as the average posterior probability of belonging to class *k* over all the individuals assigned to class *k* (van der Nest et al., 2020). When posterior probabilities are near 100%, there is minimal classification error because participants assigned to a specific trajectory have a maximal chance of belonging to that trajectory. Ideally, this probability should be near 100% for the assigned group and, conversely, near 0% for the rest, with a minimum threshold of 70% (Nagin, 2005). The average probabilities for the assigned groups are very high across indicators (79%–94%) indicating low classification error.
