# Supplementary material for: Modeling Heterogeneity in the Long-Term Trajectories of Individuals’ Well-Being
Source: Pers Soc Psychol Bull. 2025 Apr 29;52(7):2045–64. doi: 10.1177/01461672251331654 (PMC13216570; doi:10.1177/01461672251331654)
Supplement: sj-docx-1-psp-10.1177_01461672251331654 – Supplemental material for Modeling Heterogeneity in the Long-Term Trajectories of Individuals’ Well-Being [file sj-docx-1-psp-10.1177_01461672251331654.docx]

**Online supplementary materials**


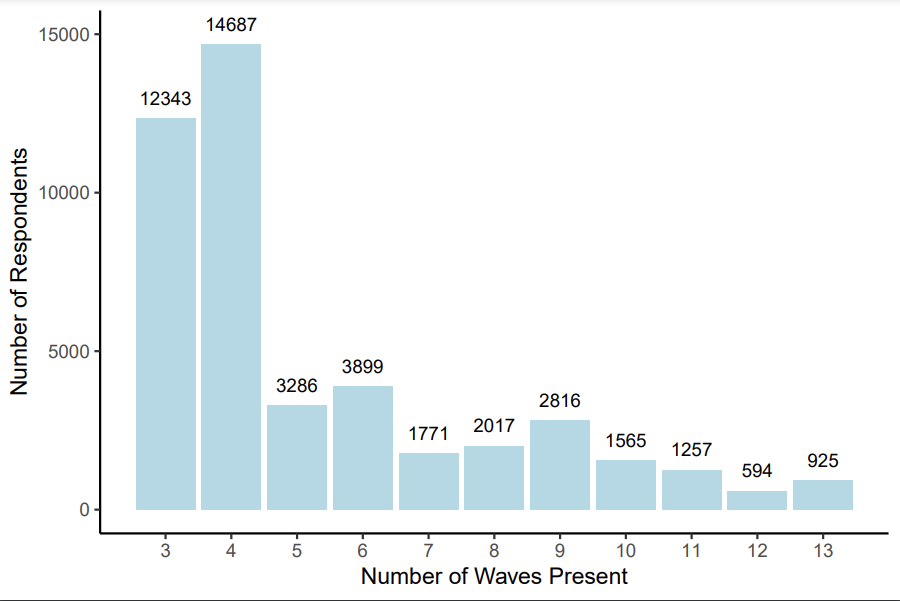


**Figure S1.** *Total number of waves completed. The figure shows the number of waves that respondents completed out of a total possible 13 waves (e.g., 12,343 participants completed 3 waves).*

**
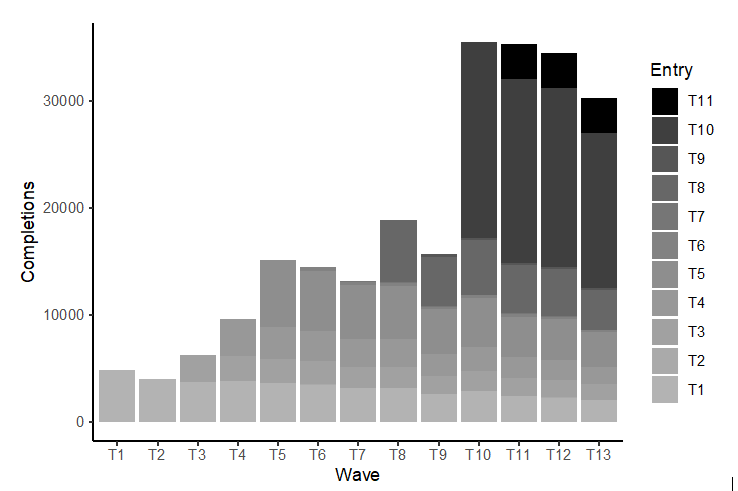
Figure S2**. *Attrition plot.*

| **Table S1.** *McDonald’s Omega coefficient for felt belongingness, social support, and self-esteem, and correlation between two items for life satisfaction* | | | | | |
| --- | --- | --- | --- | --- | --- |
| Wave | Felt Belongingness | Social Support | Self-esteem | Life Satisfaction |  |
| Wave 1 | - | - | - | - |  |
| Wave 2 | ω = .58 | ω = .75 | ω = .76 | *r* = .64* |  |
| Wave 3 | ω = .56 | ω = .82 | ω = .78 | *r* = .68* |  |
| Wave 4 | ω = .60 | ω = .79 | ω = .78 | *r* = .67* |  |
| Wave 5 | ω = .58 | ω = .81 | ω = .79 | *r* = .69* |  |
| Wave 6 | ω = .61 | ω = .81 | ω = .80 | *r* = .70* |  |
| Wave 7 | ω = .62 | ω = .83 | ω = .80 | *r* = .68* |  |
| Wave 8 | ω = .59 | ω = .81 | ω = .81 | *r* = .68* |  |
| Wave 9 | ω = .60 | ω = .83 | ω = .82 | *r* = .69* |  |
| Wave 10 | ω = .60 | ω = .81 | ω = .81 | *r* = .64* |  |
| Wave 11 | ω = .60 | ω = .84 | ω = .82 | *r* = .65* |  |
| Wave 12 | ω = .64 | ω = .83 | ω = .83 | *r* = .67* |  |
| Wave 13 | ω = .60 | ω = .84 | ω = .82 | *r* = .66* |  |

Note: ** = correlation is significant at p < .001 between both life satisfaction items.*

| **Table S2.** Average group affiliation assignment probability, conditional on assignment by maximum probability rule | | | | | | | | | |  |  |  |
| --- | --- | --- | --- | --- | --- | --- | --- | --- | --- | --- | --- | --- |
| Probability conditional on group membership | | Low | Moderate | | Moderate-high | | High | Very high | | |  |  |
| *Felt belongingness* | |  |  | |  | |  |  | | |  |  |
| Very high | | 0% | 0% | | 0% | | 8% | **85%** | | |  |  |
| High | | 0% | 0% | | 11% | | **82%** | 15% | | |  |  |
| Moderate-high | | 0% | 12% | | **83%** | | 10% | 0% | | |  |  |
| Moderate | | 11% | **86%** | | 6% | | 0% | 0% | | |  |  |
| Low | | **89%** | 2% | | 0% | | 0% | 0% | | |  |  |
| *Social Support* | |  |  | |  | |  |  | | |  |  |
| Very high | | 0% | 0% | | 0% | | 7% | **87%** | | |  |  |
| High | | 0% | 0% | | 14% | | **79%** | 13% | | |  |  |
| Moderate-high | | 0% | 12% | | **80%** | | 14% | 0% | | |  |  |
| Moderate | | 8% | **85%** | | 6% | | 0% | 0% | | |  |  |
| Low | | **92%** | 3% | | 0% | | 0% | 0% | | |  |  |
| *Self-esteem* | |  |  | |  | |  |  | | |  |  |
| Very high | | 0% | 0% | | 0% | | 5% | **87%** | | |  |  |
| High | | 0% | 0% | | 10% | | **88%** | 13% | | |  |  |
| Moderate-high | | 0% | 10% | | **84%** | | 7% | 0% | | |  |  |
| Moderate | | 8% | **87%** | | 6% | | 0% | 0% | | |  |  |
| Low | | **92%** | 3% | | 0% | | 0% | 0% | | |  |  |
| *Life satisfaction* | |  |  | |  | |  |  | | |  |  |
| Very high | | 0% | 0% | | 0% | | 4% | **89%** | | |  |  |
| High | | 0% | 0% | | 11% | | **88%** | 11% | | |  |  |
| Moderate-high | | 0% | 9% | | **84%** | | 8% | 0% | | |  |  |
| Moderate | | 6% | **89%** | | 5% | | 0% | 0% | | |  |  |
| Low | | **94%** | 2% | | 0% | | 0% | 0% | | |  |  |
|  |  | | |  | |  | | |  | | |  |

**Table S3**. Logistic models of group membership for felt belongingness.

|  | | | | | |
| --- | --- | --- | --- | --- | --- |
|  | Dependent variable: | | | | |
|  |  | | | | |
|  | Low | Moderate | Mod-High | High | Very high |
|  | (1) | (2) | (3) | (4) | (5) |
|  | | | | | |
| Age | 0.021 | -0.021^***^ | -0.007^*^ | 0.013^***^ | 0.009 |
|  | (0.016) | (0.005) | (0.003) | (0.003) | (0.005) |
|  |  |  |  |  |  |
| Age^2^ | 0.0001 | -0.001^***^ | -0.0003 | 0.001^*^ | 0.0005 |
|  | (0.001) | (0.0003) | (0.0002) | (0.0002) | (0.0003) |
|  |  |  |  |  |  |
| Age^3^ | -0.0001^*^ | 0.00003^**^ | 0.00001 | -0.00002^**^ | -0.00001 |
|  | (0.00004) | (0.00001) | (0.00001) | (0.00001) | (0.00001) |
|  |  |  |  |  |  |
| Female | -0.128 | -0.307^***^ | -0.172^**^ | 0.021 | 0.671^***^ |
|  | (0.205) | (0.088) | (0.063) | (0.061) | (0.097) |
|  |  |  |  |  |  |
| Education | -0.094^*^ | -0.041^*^ | -0.022 | 0.021 | 0.055^***^ |
|  | (0.039) | (0.016) | (0.011) | (0.011) | (0.016) |
|  |  |  |  |  |  |
| Māori | -0.111 | 0.021 | 0.109 | -0.119 | 0.023 |
|  | (0.251) | (0.110) | (0.079) | (0.078) | (0.112) |
|  |  |  |  |  |  |
| Pacific | -0.400 | 0.003 | -0.084 | 0.040 | 0.145 |
|  | (0.596) | (0.233) | (0.173) | (0.164) | (0.233) |
|  |  |  |  |  |  |
| Asian | -0.770 | 0.076 | 0.404^**^ | -0.068 | -0.849^**^ |
|  | (0.725) | (0.217) | (0.153) | (0.154) | (0.305) |
|  |  |  |  |  |  |
| Constant | -3.103^***^ | -1.254^***^ | -0.471^***^ | -0.693^***^ | -2.759^***^ |
|  | (0.288) | (0.127) | (0.094) | (0.091) | (0.144) |
|  |  |  |  |  |  |
|  | | | | | |
| Observations | 4,845 | 4,845 | 4,845 | 4,845 | 4,845 |
| Log Likelihood | -478.431 | -1,822.564 | -3,048.918 | -3,230.314 | -1,838.503 |
|  | | | | | |
| Note: | ^*^p<0.05; ^**^p<0.01; ^***^p<0.001 | | | | |

**Table S4**. Logistic models of group membership for social support.

|  | | | | | |
| --- | --- | --- | --- | --- | --- |
|  | Dependent variable: | | | | |
|  |  | | | | |
|  | Low | Moderate | Mod-High | High | Very high |
|  | (1) | (2) | (3) | (4) | (5) |
|  | | | | | |
| Age | 0.026 | 0.009 | 0.010^**^ | -0.007^*^ | -0.011^**^ |
|  | (0.014) | (0.005) | (0.003) | (0.003) | (0.004) |
|  |  |  |  |  |  |
| Age^2^ | -0.002^*^ | -0.001^**^ | 0.0001 | 0.0001 | 0.001^*^ |
|  | (0.001) | (0.0003) | (0.0002) | (0.0002) | (0.0003) |
|  |  |  |  |  |  |
| Age^3^ | 0.00000 | 0.00002 | -0.00001 | 0.00000 | -0.00001 |
|  | (0.00003) | (0.00001) | (0.00001) | (0.00001) | (0.00001) |
|  |  |  |  |  |  |
| Female | -0.505^**^ | -0.502^***^ | -0.481^***^ | 0.140^*^ | 1.042^***^ |
|  | (0.195) | (0.091) | (0.063) | (0.063) | (0.088) |
|  |  |  |  |  |  |
| Education | -0.181^***^ | -0.068^***^ | -0.033^**^ | 0.015 | 0.096^***^ |
|  | (0.039) | (0.017) | (0.012) | (0.011) | (0.014) |
|  |  |  |  |  |  |
| Māori | -0.100 | 0.236^*^ | 0.119 | -0.210^**^ | -0.015 |
|  | (0.243) | (0.113) | (0.080) | (0.080) | (0.098) |
|  |  |  |  |  |  |
| Pacific | 0.075 | 0.392 | 0.104 | -0.273 | -0.063 |
|  | (0.473) | (0.224) | (0.170) | (0.175) | (0.215) |
|  |  |  |  |  |  |
| Asian | 0.199 | 1.087^***^ | 0.445^**^ | -0.585^***^ | -0.915^***^ |
|  | (0.527) | (0.185) | (0.155) | (0.173) | (0.245) |
|  |  |  |  |  |  |
| Constant | -2.323^***^ | -1.407^***^ | -0.403^***^ | -0.733^***^ | -2.688^***^ |
|  | (0.262) | (0.132) | (0.094) | (0.094) | (0.129) |
|  |  |  |  |  |  |
|  | | | | | |
| Observations | 4,845 | 4,845 | 4,845 | 4,845 | 4,845 |
| Log Likelihood | -498.297 | -1,705.981 | -3,015.447 | -3,090.020 | -2,244.297 |
|  | | | | | |
| Note: | ^*^p<0.05; ^**^p<0.01; ^***^p<0.001 | | | | |

**Table S5**. Logistic models of group membership for self-esteem.

|  | | | | | |
| --- | --- | --- | --- | --- | --- |
|  | Dependent variable: | | | | |
|  |  | | | | |
|  | Low | Moderate | Mod-High | High | Very high |
|  | (1) | (2) | (3) | (4) | (5) |
|  | | | | | |
| Age | -0.028^**^ | -0.029^***^ | -0.003 | 0.014^***^ | 0.025^***^ |
|  | (0.011) | (0.004) | (0.003) | (0.003) | (0.006) |
|  |  |  |  |  |  |
| Age^2^ | -0.001 | -0.001^**^ | 0.0001 | 0.0004 | -0.0002 |
|  | (0.001) | (0.0003) | (0.0002) | (0.0002) | (0.0004) |
|  |  |  |  |  |  |
| Age^3^ | -0.00000 | 0.00003^***^ | 0.00000 | -0.00001^*^ | -0.00001 |
|  | (0.00002) | (0.00001) | (0.00001) | (0.00001) | (0.00001) |
|  |  |  |  |  |  |
| Female | 0.126 | 0.199^*^ | 0.047 | -0.209^***^ | 0.146 |
|  | (0.168) | (0.086) | (0.065) | (0.060) | (0.100) |
|  |  |  |  |  |  |
| Education | -0.136^***^ | -0.050^**^ | -0.036^**^ | 0.049^***^ | 0.065^***^ |
|  | (0.031) | (0.015) | (0.012) | (0.011) | (0.018) |
|  |  |  |  |  |  |
| Māori | -0.270 | -0.240^*^ | 0.069 | -0.003 | 0.272^*^ |
|  | (0.202) | (0.107) | (0.081) | (0.077) | (0.121) |
|  |  |  |  |  |  |
| Pacific | -0.681 | -0.052 | -0.003 | -0.002 | 0.286 |
|  | (0.518) | (0.218) | (0.175) | (0.164) | (0.253) |
|  |  |  |  |  |  |
| Asian | -0.339 | -0.202 | -0.270 | 0.331^*^ | 0.070 |
|  | (0.467) | (0.219) | (0.177) | (0.151) | (0.246) |
|  |  |  |  |  |  |
| Constant | -2.346^***^ | -1.301^***^ | -0.746^***^ | -0.597^***^ | -2.807^***^ |
|  | (0.226) | (0.122) | (0.097) | (0.090) | (0.155) |
|  |  |  |  |  |  |
|  | | | | | |
| Observations | 4,845 | 4,845 | 4,845 | 4,845 | 4,845 |
| Log Likelihood | -691.908 | -2,017.496 | -2,925.430 | -3,264.345 | -1,566.043 |
|  | | | | | |
| Note: | ^*^p<0.05; ^**^p<0.01; ^***^p<0.001 | | | | |

**Table S6**. Logistic models of group membership for life satisfaction.

|  | | | | | |
| --- | --- | --- | --- | --- | --- |
|  | Dependent variable: | | | | |
|  |  | | | | |
|  | Low | Moderate | Mod-High | High | Very high |
|  | (1) | (2) | (3) | (4) | (5) |
|  | | | | | |
| Age | 0.029^*^ | -0.014^**^ | -0.005 | 0.010^**^ | 0.006 |
|  | (0.014) | (0.005) | (0.003) | (0.003) | (0.005) |
|  |  |  |  |  |  |
| Age^2^ | -0.001^*^ | -0.001^**^ | 0.0002 | 0.0003 | 0.0004 |
|  | (0.001) | (0.0003) | (0.0002) | (0.0002) | (0.0003) |
|  |  |  |  |  |  |
| Age^3^ | -0.0001^*^ | 0.00001 | -0.00001 | -0.00001 | 0.00000 |
|  | (0.00004) | (0.00001) | (0.00001) | (0.00001) | (0.00001) |
|  |  |  |  |  |  |
| Female | -0.139 | -0.141 | -0.172^**^ | 0.098 | 0.387^***^ |
|  | (0.168) | (0.093) | (0.064) | (0.060) | (0.105) |
|  |  |  |  |  |  |
| Education | -0.159^***^ | -0.081^***^ | -0.017 | 0.047^***^ | 0.055^**^ |
|  | (0.033) | (0.017) | (0.012) | (0.011) | (0.018) |
|  |  |  |  |  |  |
| Māori | -0.017 | 0.306^**^ | 0.027 | -0.171^*^ | -0.010 |
|  | (0.197) | (0.108) | (0.081) | (0.076) | (0.130) |
|  |  |  |  |  |  |
| Pacific | -0.549 | 0.102 | 0.166 | -0.242 | 0.291 |
|  | (0.518) | (0.238) | (0.168) | (0.165) | (0.253) |
|  |  |  |  |  |  |
| Asian | -0.087 | -0.063 | 0.226 | 0.046 | -0.790^*^ |
|  | (0.469) | (0.251) | (0.157) | (0.150) | (0.348) |
|  |  |  |  |  |  |
| Constant | -2.203^***^ | -1.369^***^ | -0.615^***^ | -0.611^***^ | -2.945^***^ |
|  | (0.228) | (0.132) | (0.095) | (0.090) | (0.159) |
|  |  |  |  |  |  |
|  | | | | | |
| Observations | 4,845 | 4,845 | 4,845 | 4,845 | 4,845 |
| Log Likelihood | -646.309 | -1,695.455 | -2,976.017 | -3,301.329 | -1,530.749 |
|  | | | | | |
| Note: | ^*^p<0.05; ^**^p<0.01; ^***^p<0.001 | | | | |

**Table S7**. Logistic models of group membership for felt belongingness (without education).

|  | | | | | |
| --- | --- | --- | --- | --- | --- |
|  | Dependent variable: | | | | |
|  |  | | | | |
|  | Low | Moderate | Mod-High | High | Very high |
|  | (1) | (2) | (3) | (4) | (5) |
|  | | | | | |
| Age | -0.023^***^ | -0.016^***^ | -0.004^**^ | 0.011^***^ | 0.014^***^ |
|  | (0.004) | (0.002) | (0.001) | (0.001) | (0.002) |
|  |  |  |  |  |  |
| Age^2^ | -0.001^***^ | -0.0001^*^ | 0.0001 | -0.00004 | -0.0001 |
|  | (0.0002) | (0.0001) | (0.00005) | (0.00004) | (0.0001) |
|  |  |  |  |  |  |
| Age^3^ | -0.00002^*^ | -0.00000 | 0.00000 | -0.00000 | 0.00000 |
|  | (0.00001) | (0.00000) | (0.00000) | (0.00000) | (0.00000) |
|  |  |  |  |  |  |
| Female | -0.349^***^ | -0.266^***^ | -0.215^***^ | 0.143^***^ | 0.650^***^ |
|  | (0.053) | (0.028) | (0.021) | (0.020) | (0.035) |
|  |  |  |  |  |  |
| Māori | 0.191^*^ | 0.123^**^ | 0.076^*^ | -0.146^***^ | -0.052 |
|  | (0.075) | (0.040) | (0.032) | (0.031) | (0.048) |
|  |  |  |  |  |  |
| Pacific | 0.165 | 0.038 | 0.023 | -0.102 | 0.093 |
|  | (0.156) | (0.086) | (0.067) | (0.065) | (0.098) |
|  |  |  |  |  |  |
| Asian | 0.098 | 0.191^**^ | 0.219^***^ | -0.224^***^ | -0.357^***^ |
|  | (0.118) | (0.062) | (0.049) | (0.050) | (0.090) |
|  |  |  |  |  |  |
| Constant | -3.061^***^ | -1.569^***^ | -0.665^***^ | -0.518^***^ | -2.547^***^ |
|  | (0.049) | (0.025) | (0.020) | (0.020) | (0.035) |
|  |  |  |  |  |  |
|  | | | | | |
| Observations | 44,632 | 44,632 | 44,632 | 44,632 | 44,632 |
| Log Likelihood | -6,554.079 | -18,558.600 | -27,762.970 | -29,715.340 | -15,213.730 |
|  | | | | | |
| Note: | ^*^p<0.05; ^**^p<0.01; ^***^p<0.001 | | | | |

**Table S8**. Logistic models of group membership, social support (without education).

|  | | | | | |
| --- | --- | --- | --- | --- | --- |
|  | Dependent variable: | | | | |
|  |  | | | | |
|  | Low | Moderate | Mod-High | High | Very high |
|  | (1) | (2) | (3) | (4) | (5) |
|  | | | | | |
| Age | 0.006 | 0.003 | 0.004^**^ | 0.002 | -0.009^***^ |
|  | (0.004) | (0.002) | (0.001) | (0.001) | (0.001) |
|  |  |  |  |  |  |
| Age^2^ | -0.001^***^ | -0.0003^***^ | 0.0002^***^ | -0.00000 | -0.0001 |
|  | (0.0002) | (0.0001) | (0.00005) | (0.00004) | (0.0001) |
|  |  |  |  |  |  |
| Age^3^ | -0.00003^**^ | -0.00000 | -0.00000 | -0.00000 | 0.00001^***^ |
|  | (0.00001) | (0.00000) | (0.00000) | (0.00000) | (0.00000) |
|  |  |  |  |  |  |
| Female | -0.570^***^ | -0.524^***^ | -0.486^***^ | 0.200^***^ | 0.879^***^ |
|  | (0.058) | (0.031) | (0.021) | (0.021) | (0.028) |
|  |  |  |  |  |  |
| Māori | 0.305^***^ | 0.175^***^ | 0.036 | -0.075^*^ | -0.102^**^ |
|  | (0.082) | (0.047) | (0.032) | (0.031) | (0.037) |
|  |  |  |  |  |  |
| Pacific | 0.059 | 0.290^**^ | -0.012 | -0.051 | -0.104 |
|  | (0.188) | (0.094) | (0.068) | (0.065) | (0.079) |
|  |  |  |  |  |  |
| Asian | 0.487^***^ | 0.676^***^ | 0.263^***^ | -0.352^***^ | -0.522^***^ |
|  | (0.120) | (0.064) | (0.050) | (0.053) | (0.068) |
|  |  |  |  |  |  |
| Constant | -3.171^***^ | -1.858^***^ | -0.559^***^ | -0.719^***^ | -1.915^***^ |
|  | (0.052) | (0.029) | (0.020) | (0.020) | (0.027) |
|  |  |  |  |  |  |
|  | | | | | |
| Observations | 44,632 | 44,632 | 44,632 | 44,632 | 44,632 |
| Log Likelihood | -5,621.476 | -14,773.330 | -27,356.120 | -28,867.140 | -21,989.300 |
|  | | | | | |
| Note: | ^*^p<0.05; ^**^p<0.01; ^***^p<0.001 | | | | |

**Table S9**. Logistic models of group membership, self-esteem (without education).

|  | | | | | |
| --- | --- | --- | --- | --- | --- |
|  | Dependent variable: | | | | |
|  |  | | | | |
|  | Low | Moderate | Mod-High | High | Very high |
|  | (1) | (2) | (3) | (4) | (5) |
|  | | | | | |
| Age | -0.038^***^ | -0.022^***^ | -0.005^***^ | 0.014^***^ | 0.031^***^ |
|  | (0.003) | (0.002) | (0.001) | (0.001) | (0.002) |
|  |  |  |  |  |  |
| Age^2^ | -0.0004^*^ | 0.00002 | 0.0001 | -0.0003^***^ | -0.0003^***^ |
|  | (0.0001) | (0.0001) | (0.00005) | (0.00005) | (0.0001) |
|  |  |  |  |  |  |
| Age^3^ | -0.00002^**^ | 0.00000 | 0.00000^*^ | 0.00000 | -0.00001^**^ |
|  | (0.00001) | (0.00000) | (0.00000) | (0.00000) | (0.00000) |
|  |  |  |  |  |  |
| Female | 0.072 | 0.169^***^ | 0.084^***^ | -0.198^***^ | 0.048 |
|  | (0.048) | (0.027) | (0.022) | (0.020) | (0.033) |
|  |  |  |  |  |  |
| Māori | 0.038 | -0.030 | 0.030 | -0.091^**^ | 0.190^***^ |
|  | (0.067) | (0.040) | (0.032) | (0.031) | (0.048) |
|  |  |  |  |  |  |
| Pacific | -0.247 | -0.005 | -0.003 | -0.027 | 0.211^*^ |
|  | (0.154) | (0.082) | (0.068) | (0.065) | (0.102) |
|  |  |  |  |  |  |
| Asian | -0.387^***^ | -0.200^**^ | -0.087 | 0.153^**^ | 0.337^***^ |
|  | (0.117) | (0.065) | (0.053) | (0.049) | (0.076) |
|  |  |  |  |  |  |
| Constant | -3.073^***^ | -1.706^***^ | -0.925^***^ | -0.277^***^ | -2.342^***^ |
|  | (0.047) | (0.026) | (0.021) | (0.019) | (0.033) |
|  |  |  |  |  |  |
|  | | | | | |
| Observations | 44,632 | 44,632 | 44,632 | 44,632 | 44,632 |
| Log Likelihood | -8,124.277 | -19,783.190 | -27,080.720 | -29,583.740 | -14,055.230 |
|  | | | | | |
| Note: | ^*^p<0.05; ^**^p<0.01; ^***^p<0.001 | | | | |

**Table S10**. Logistic models of group membership, life satisfaction (without education).

|  | | | | | |
| --- | --- | --- | --- | --- | --- |
|  | Dependent variable: | | | | |
|  |  | | | | |
|  | Low | Moderate | Mod-High | High | Very high |
|  | (1) | (2) | (3) | (4) | (5) |
|  | | | | | |
| Age | -0.014^***^ | -0.013^***^ | -0.006^***^ | 0.008^***^ | 0.016^***^ |
|  | (0.004) | (0.002) | (0.001) | (0.001) | (0.002) |
|  |  |  |  |  |  |
| Age^2^ | -0.001^***^ | -0.0001^*^ | 0.0001 | -0.0001^**^ | 0.00004 |
|  | (0.0002) | (0.0001) | (0.00005) | (0.00004) | (0.0001) |
|  |  |  |  |  |  |
| Age^3^ | -0.00003^***^ | -0.00001^*^ | -0.00000 | 0.00000 | 0.00000 |
|  | (0.00001) | (0.00000) | (0.00000) | (0.00000) | (0.00000) |
|  |  |  |  |  |  |
| Female | -0.379^***^ | -0.200^***^ | -0.118^***^ | 0.078^***^ | 0.468^***^ |
|  | (0.055) | (0.030) | (0.022) | (0.020) | (0.036) |
|  |  |  |  |  |  |
| Māori | 0.317^***^ | 0.370^***^ | 0.055 | -0.229^***^ | -0.100 |
|  | (0.076) | (0.042) | (0.032) | (0.030) | (0.052) |
|  |  |  |  |  |  |
| Pacific | 0.286 | 0.281^**^ | 0.114 | -0.242^***^ | -0.068 |
|  | (0.156) | (0.088) | (0.067) | (0.063) | (0.111) |
|  |  |  |  |  |  |
| Asian | -0.062 | 0.259^***^ | 0.042 | -0.061 | -0.298^**^ |
|  | (0.134) | (0.067) | (0.052) | (0.048) | (0.094) |
|  |  |  |  |  |  |
| Constant | -3.170^***^ | -1.920^***^ | -0.841^***^ | -0.141^***^ | -2.609^***^ |
|  | (0.051) | (0.028) | (0.021) | (0.019) | (0.036) |
|  |  |  |  |  |  |
|  | | | | | |
| Observations | 44,632 | 44,632 | 44,632 | 44,632 | 44,632 |
| Log Likelihood | -6,114.746 | -16,041.170 | -26,814.780 | -30,699.020 | -13,762.700 |
|  | | | | | |
| Note: | ^*^p<0.05; ^**^p<0.01; ^***^p<0.001 | | | | |
