# Supplementary figures and images for: Modeling Heterogeneity in the Long-Term Trajectories of Individuals’ Well-Being
Source: Pers Soc Psychol Bull. 2025 Apr 29;52(7):2045–64. doi: 10.1177/01461672251331654 (PMC13216570; doi:10.1177/01461672251331654)

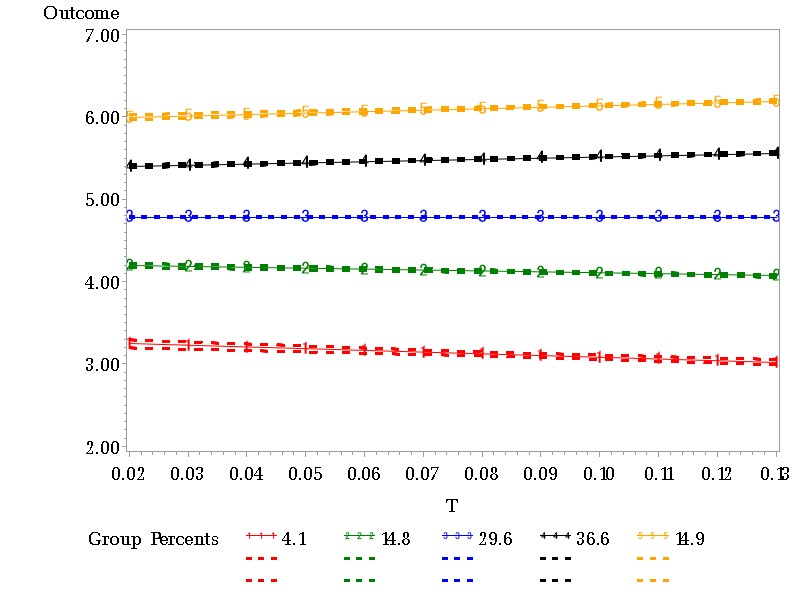

Supplement: sj-zip-2-psp-10.1177_01461672251331654 – Supplemental material for Modeling Heterogeneity in the Long-Term Trajectories of Individuals’ Well-Being [file sj-zip-2-psp-10.1177_01461672251331654.zip › OSF/OSF/FB_Multi.jpg]

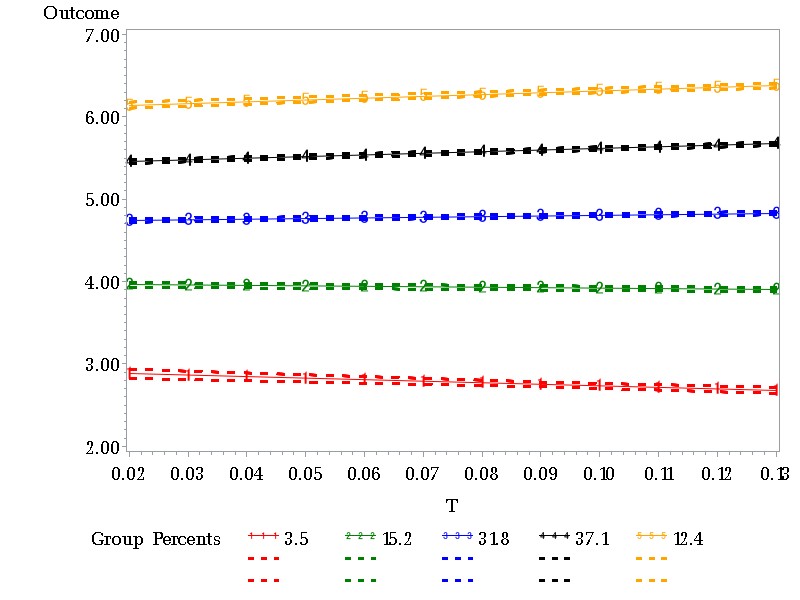

Supplement: sj-zip-2-psp-10.1177_01461672251331654 – Supplemental material for Modeling Heterogeneity in the Long-Term Trajectories of Individuals’ Well-Being [file sj-zip-2-psp-10.1177_01461672251331654.zip › OSF/OSF/FB_Trajectories.jpg]

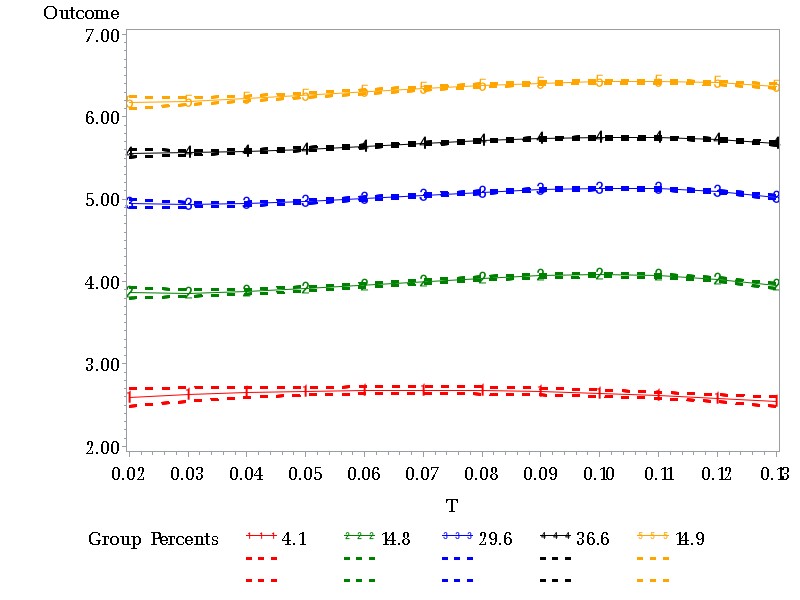

Supplement: sj-zip-2-psp-10.1177_01461672251331654 – Supplemental material for Modeling Heterogeneity in the Long-Term Trajectories of Individuals’ Well-Being [file sj-zip-2-psp-10.1177_01461672251331654.zip › OSF/OSF/LS_Multi.jpg]

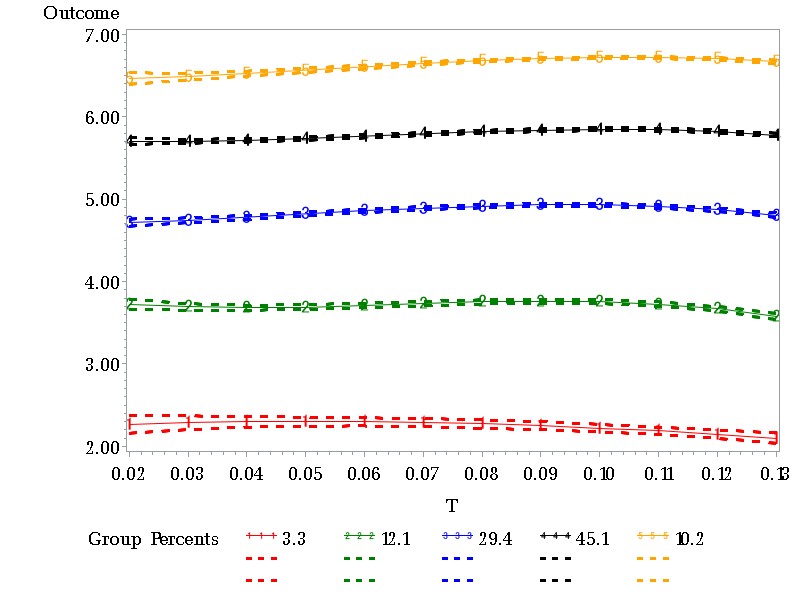

Supplement: sj-zip-2-psp-10.1177_01461672251331654 – Supplemental material for Modeling Heterogeneity in the Long-Term Trajectories of Individuals’ Well-Being [file sj-zip-2-psp-10.1177_01461672251331654.zip › OSF/OSF/LS_Trajectories.jpg]

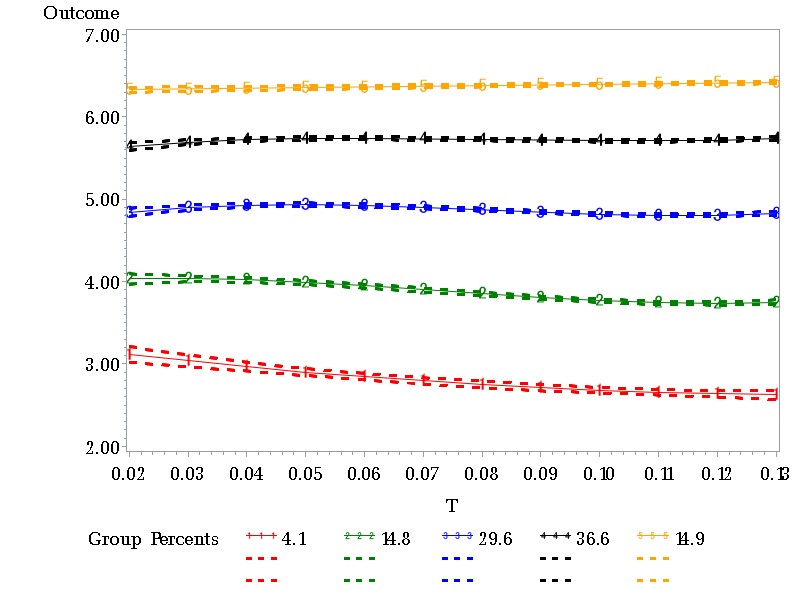

Supplement: sj-zip-2-psp-10.1177_01461672251331654 – Supplemental material for Modeling Heterogeneity in the Long-Term Trajectories of Individuals’ Well-Being [file sj-zip-2-psp-10.1177_01461672251331654.zip › OSF/OSF/SE_Multi.jpg]

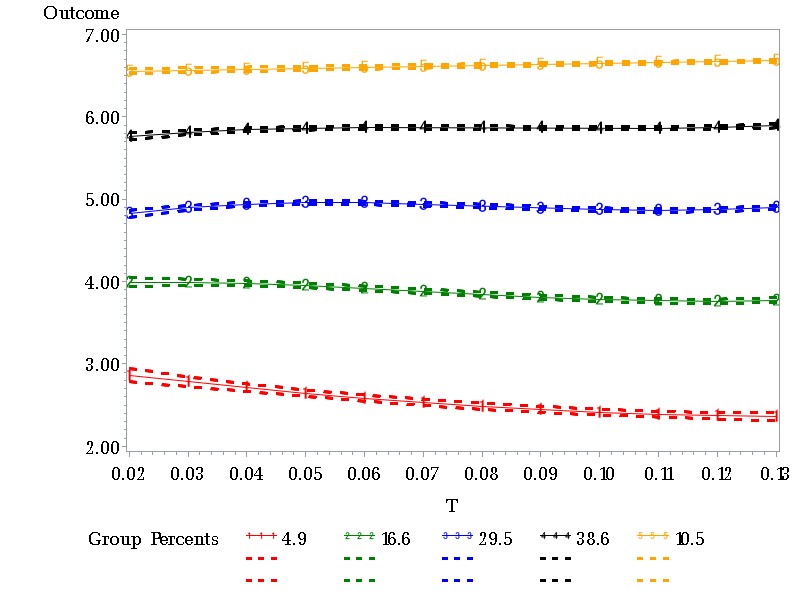

Supplement: sj-zip-2-psp-10.1177_01461672251331654 – Supplemental material for Modeling Heterogeneity in the Long-Term Trajectories of Individuals’ Well-Being [file sj-zip-2-psp-10.1177_01461672251331654.zip › OSF/OSF/SE_Trajectories.jpg]

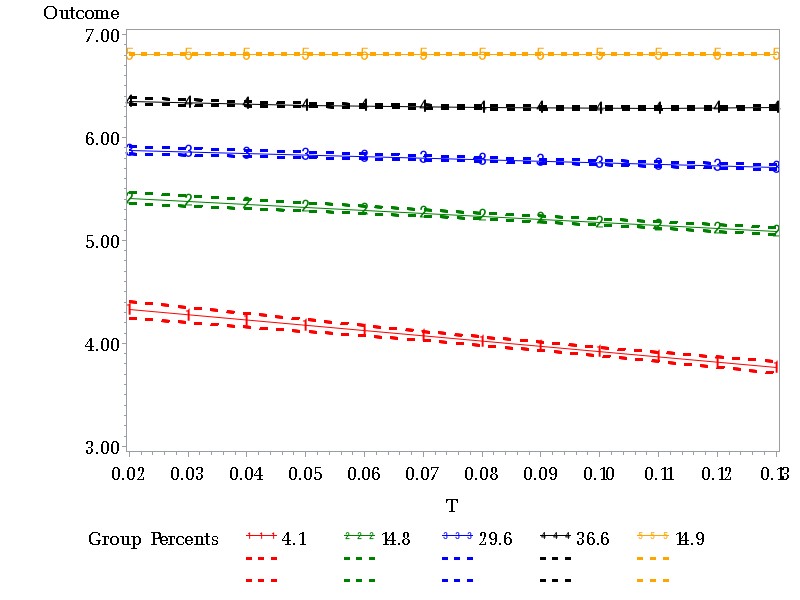

Supplement: sj-zip-2-psp-10.1177_01461672251331654 – Supplemental material for Modeling Heterogeneity in the Long-Term Trajectories of Individuals’ Well-Being [file sj-zip-2-psp-10.1177_01461672251331654.zip › OSF/OSF/SS_Multi.jpg]

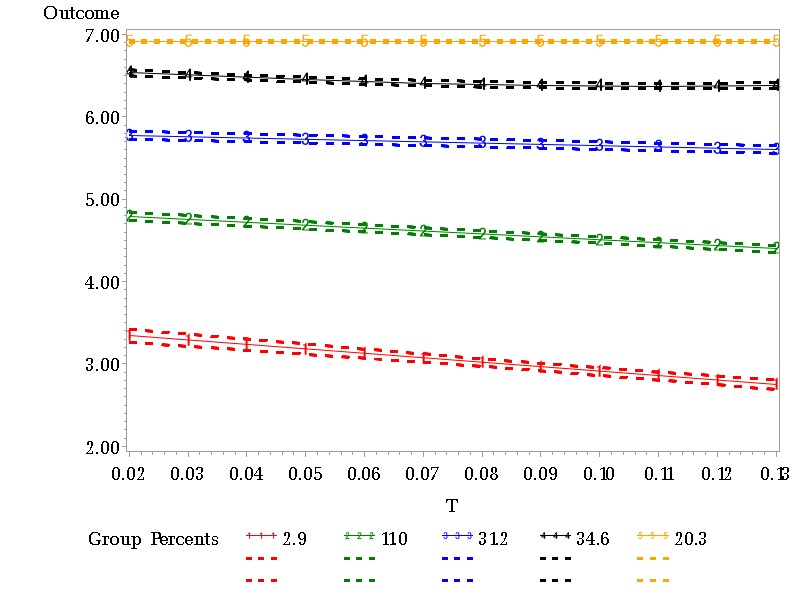

Supplement: sj-zip-2-psp-10.1177_01461672251331654 – Supplemental material for Modeling Heterogeneity in the Long-Term Trajectories of Individuals’ Well-Being [file sj-zip-2-psp-10.1177_01461672251331654.zip › OSF/OSF/SS_Trajectories.jpg]
